# Contingent evolution of alternative metabolic network topologies determines whether cross-feeding evolves

**DOI:** 10.1038/s42003-020-1107-x

**Published:** 2020-07-29

**Authors:** Jeroen Meijer, Bram van Dijk, Paulien Hogeweg

**Affiliations:** Theoretical Biology and Bioinformatics, Department of Biology, Utrecht University, Padualaan 8, Utrecht, 3584 CH The Netherlands

**Keywords:** Computational models, Evolution, Microbial ecology, Evolutionary ecology, Emergence

## Abstract

Metabolic exchange is widespread in natural microbial communities and an important driver of ecosystem structure and diversity, yet it remains unclear what determines whether microbes evolve division of labor or maintain metabolic autonomy. Here we use a mechanistic model to study how metabolic strategies evolve in a constant, one resource environment, when metabolic networks are allowed to freely evolve. We find that initially identical ancestral communities of digital organisms follow different evolutionary trajectories, as some communities become dominated by a single, autonomous lineage, while others are formed by stably coexisting lineages that cross-feed on essential building blocks. Our results show how without presupposed cellular trade-offs or external drivers such as temporal niches, diverse metabolic strategies spontaneously emerge from the interplay between ecology, spatial structure, and metabolic constraints that arise during the evolution of metabolic networks. Thus, in the long term, whether microbes remain autonomous or evolve metabolic division of labour is an evolutionary contingency.

## Abstract (web summary)

Using a mechanistic individual-based model, Meijer et al. show that diverse metabolic strategies emerge through the interplay between evolved metabolic constraints and ecology, without preconditions that assume cellular trade-offs or external drivers for metabolic division of labour.

## Introduction

Natural microbial communities are typically complex, composed of many different taxonomic groups that stably coexist. The complexity of these communities might partly reflect the complexity of their environment, which can generate and maintain diversity by allowing specialisation on different niches^1–10^. Experimental evolution has shown how initially clonal populations can adaptively diversify into stably coexisting ecotypes, each specialised on pre-existing niches defined by available nutrients^1^, spatial structure^2,11^, temporal variability such as the feast and famine cycles in serial transfer^3–9^, or combinations thereof^10^. However, even in constant, unstructured environments with a single limiting carbon source metabolic diversification routinely evolves^4,12–14^. Here, new niches are constructed by microbes themselves, as metabolic byproducts released by one become the growth substrate for another, allowing stable coexistence mediated by metabolic interactions. For example, initially clonal *Escherichia coli* populations grown in a glucose-limited chemostat genetically diversify into a lineage that rapidly but inefficiently grows on the provided resource, and lineages that specialise in using the overflow acetate produced by the first lineage^4,13,15^. Niche construction can thus lead to stable coexistence even with a single resource, which according to the competitive exclusion principle^16^ would support only a single species.

Several mechanisms have been proposed that might explain both the evolution of cross-feeding in simple, constant environments as well as the prevalence of cross-feeding in natural communities^17–22^ (see ref. ^23^ for a review). For example, cellular or metabolic trade-offs might favour metabolic specialisation^24,25^, and division of labour might increase productivity of a community^26^. Alternatively, the “Black Queen Hypothesis” holds that gene loss can be adaptive for functions that are costly, leaky and essential—such as the production of essential building blocks that end up in the environment through diffusion or lysis—provided other community members compensate for the lost function^27–29^. Indeed, by removing two metabolic genes in *E.coli*, a recent study showed that in a synthetic community with engineered obligate dependencies for amino acids, strains grow up to 20% faster compared with the autonomous wild-type, suggesting positive selection for the loss of biosynthetic genes^18^. Cross-feeding might thus be caused by a gene loss ratchet in a community of initially autonomous microbes that, driven by escaping the burden of biosynthesis genes, evolve complementary metabolic networks and become dependent^30,31^.

Theoretical studies on the evolution of metabolic dependency typically predefine a limited number of metabolic strategies^32^, take cellular trade-offs for granted^25,32–36^, or explicitly assume external forcing such as seasonality^37,38^, and investigate how other conditions favour autonomous or cross-feeding strategies, or prevent the evolution of metabolic free-loaders^39^. For example, by constructing genome-scale metabolic models and limiting the number of reactions that can be performed by a single individual, under certain conditions multiple cross-feeding strategies together can outperform an autonomous strategy^26^. However, these trade-offs are not always necessarily an inescapable physical reality that must be faced, but are themselves evolved cellular properties^40^, and little attention is given to the evolution *of* trade-offs compared with the evolution *on* trade-offs.

In addition, these models tend to assume ecological and evolutionary timescales are separated—the fate of each individual mutation is determined by playing out ecological dynamics before the next mutation arrives. We know from experimental studies^9,41–45^ that microbial communities do not operate in such a mutation-limited domain, and instead, multiple mutations continuously emerge and cause complex evolutionary dynamics and contingency. Theoretical work^46,47^ demonstrated that under such true “eco-evolutionary” conditions, evolutionary dynamics qualitatively change and result in more complex and diverse ecosystems.

Here, we investigate how different metabolic strategies can evolve using a bottom-up, mechanistic model of microbial eco-evolutionary dynamics that explicitly accounts for the high diversity in communities without assuming cellular trade-offs or external drivers for metabolic division of labour. We show that initially identical populations—when propagated under identical conditions—can reach two qualitatively different eco-evolutionary attractors: a community of cross-feeding metabolic specialists or, alternatively, a community formed by a single lineage of microbes that are metabolically autonomous, producing all metabolites they need. Which type of community evolves is dependent on, and can be predicted from, a “frozen metabolic accident”: the topology of the metabolic network that typically fixes much earlier in evolution. Differences between these topologies appear to be neutral across populations at time of fixation, but have far reaching repercussions for subsequent evolution.

## Results

### Model overview

See “Methods” for a detailed model description and parameters.

**Fig. 1:**
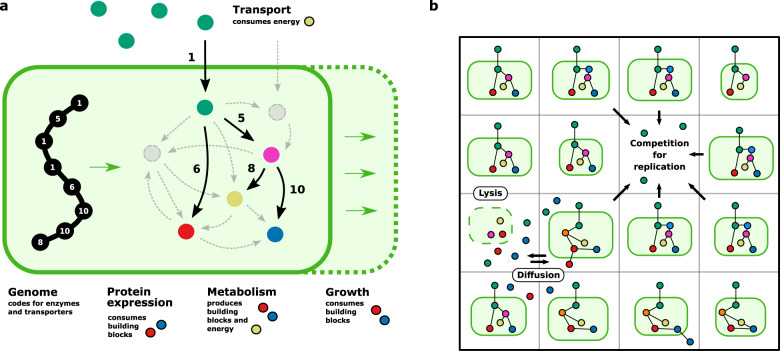
**Model of microbial eco-evolutionary dynamics**. **a** Genes on a linear genome code for specific metabolic enzymes that catalyse individual reactions of the metabolic network. To express proteins and grow, microbes require two non-substitutable building block metabolites *B*_1_ and *B*_2_ (red, blue) that do not natively occur in their environment, but can be metabolised from the single provided resource *R* (green) by expressing the right metabolic pathways. Active transport of metabolites across the cell membrane requires an energy metabolite *E* (yellow). The genome of a single microbe typically covers a small subset of the complete “chemical universe” of 59 reactions (see Methods). **b** Microbes compete for space and metabolites on a 45 × 45 lattice. They can reproduce in an adjacent empty space if they meet the minimal division cell size. Here, microbes NE and W of the empty space are too small to reproduce. Upon replication, genomes can mutate through gene duplication and deletion, discovery of new genes, and point mutations that can change the expression rate and kinetic parameters of individual genes. New genes can also be acquired via horizontal gene transfer from nearby microbes. Active transport of metabolites and lysis changes the composition of a microbes’ local environment.

In the model, we explicitly incorporate a “chemical universe”, metabolism, cell growth and division, genome evolution and a two-dimensional spatial environment that all co-evolve (Fig. 1). We do not predefine fitness (such as a target genotype or biomass reaction to be optimised), but set basic rules for cell growth, reproduction and death. This means whether a mutation is beneficial, neutral or deleterious depends on local environmental conditions and interactions, cellular state and genomic background, all of which are shaped by prior evolution in the model. As a consequence, metabolic or ecological strategies are not predefined, but emerge during evolutionary simulations as microbes evolve and explore the possibilities of the chemical universe and reshape their local environment by metabolite uptake and exchange. This approach allows us to de novo create microbial communities with their own evolutionary histories and study them with access to a perfect digital “fossil record”.

Microbes compete for a single resource molecule *R* and limited space on a 2d grid, reproducing locally into empty neighbouring sites (Fig. 1a, b). They require two essential (non-substitutable) building block metabolites *B*_1_, *B*_2_ for cell growth and expressing proteins that perform metabolic functions. Building blocks do not natively occur in the environment, but can be synthesised from the provided resource by expressing relevant metabolic proteins. In addition to building blocks, microbes require energy metabolite *E* to operate transporter proteins to pump metabolites (such as the provided resource) in and out of the cell. The chemical universe available for evolution to meet these metabolic demands consists of a predefined set of nine metabolites (*R*, *B*_1_, *B*_2_, *M*_1−5_ and *E*) connected by 59 reactions (43 conversion reactions and 16 transport reactions, see Methods), that contains many redundant pathways and provides many degrees of freedom to form functional metabolic networks.

Proteins catalyse individual reactions (e.g. 1*R* → 1*M*_1_ + 5*E*) that can be combined to form metabolic pathways. They are coded on the microbe’s genome, which typically covers only a small subset of all reactions. When cells reproduce the genome can mutate, allowing the metabolic networks to evolve by tuning the rates of individual reactions through point mutations (basal expression rate and kinetic parameters of the enzyme) and gene copy number (gene deletion, duplication). New pathways can be formed by discovering new genes or through horizontal gene transfer from nearby cells.

Cells can reproduce in a neighbouring empty site if they meet a minimal division size (Fig. 1b), with competition biased towards larger cells when multiple cells are eligible. Cell death is modelled as a stochastic process with a basal death rate that is potentially elevated when internal metabolites reach toxic concentrations. Cell lysis releases all internal metabolites into the local environment, which then locally diffuse and become available for other nearby microbes to take up. In this way, microbes change the metabolite composition of their local environment through active transport, passive diffusion across the cell membrane and cell death (see Fig. 1b). Motivated by experimental work^11,48^ that shows that microscale gradients quickly establish and influence microbial metabolism and community dynamics, we first consider evolution in a spatially structured environment with limited diffusion (mimicking biofilm conditions), and subsequently investigate evolution simulating a well-mixed medium.

We constructed an initial population of “minimally viable” microbes by generating 2025 randomly parameterised genomes coding for metabolic networks that contain a food importer and randomly selected genes to produce both building blocks. We then evolved 60 identical copies of this population in parallel under the exact same conditions for 10^6^ time steps (~4 × 10^5^ generations), while fluxing in food metabolite *R* at a constant rate at all grid points. Using this model, we examine whether “ecosystem based” metabolic strategies evolve, i.e. cross-feeding species with complementary metabolic networks, or “individual-based” strategies in the form of autonomous microbes that produce all required building blocks.

### Diverse metabolic strategies evolve in a simple, constant environment

We investigated the evolution of metabolic strategies with a mechanistic model, first focusing on the effect of contingency with a parallel evolution experiment. The ancestral community consists of microbes with metabolic networks composed of a food importer and randomly selected genes to produce both building blocks, all of which have randomly sampled kinetic parameters and expression rates. During the simulations point mutations fix that tune fluxes through specific reactions, and metabolic networks are extended with reactions that are dedicated to producing energy—which allows increased food uptake—and reactions that process byproducts for more energy and/or building blocks. Furthermore, importers are recruited to recycle building blocks that accumulate in the environment through cell lysis. Thus eventually, all populations evolve efficient, closed metabolic networks that make use of all produced metabolites.

**Fig. 2:**
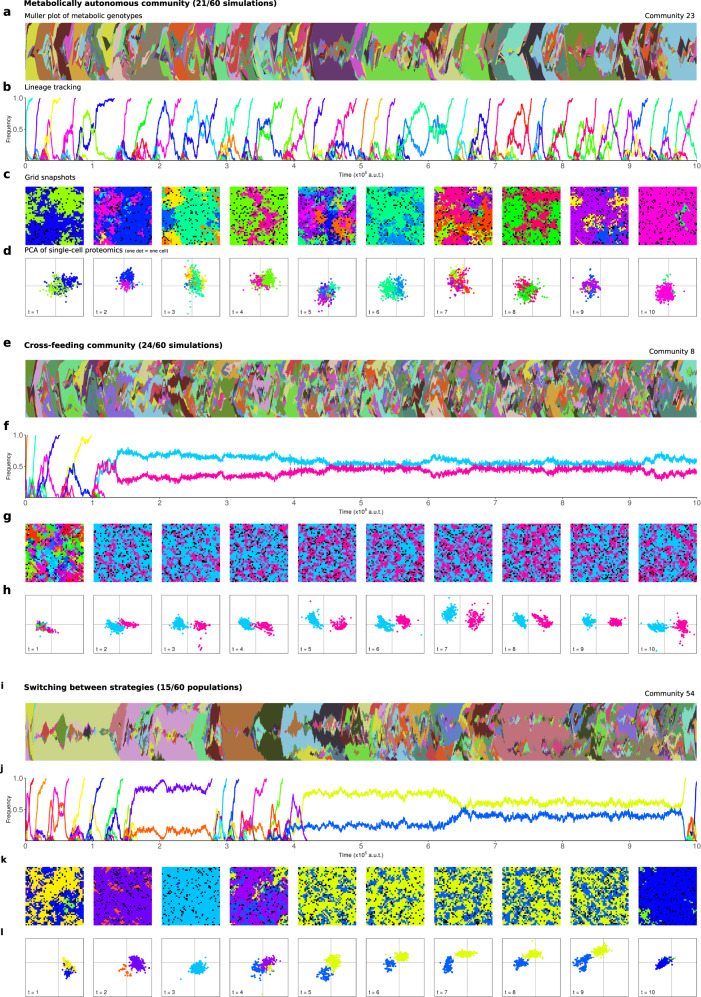
**Emergence of diverse metabolic strategies**. **a**–**d** Example of population dominated by a single autonomous lineage. **a** Muller plot showing relative frequencies and phylogenetic relationships of different metabolic genotypes throughout the experiment. Clades of microbes with different metabolic genotypes (colours) continuously evolve, resulting in complex evolutionary dynamics of competition and leapfrogging. **b** Tracking ancestral relationships with renewing lineage markers shows a continued turnover in markers, indicating that at any point during the simulation all microbes have a recent common ancestor. **c** Snapshots of spatial environment. **d** Principal component analysis of single-cell proteomes shows that in these communities all microbes express similar proteins. **e**–**h** Example of a population that diversifies in two lineages that cross-feed on essential building blocks. **g** Lineages form an interleaved pattern in the spatial environment (see Supplementary Fig. 6 and Supplementary Movie 1). **h** Single-cell proteomes show that these lineages express different metabolic enzymes. **i**–**l** Example of a population that switches between autonomous and cross-feeding strategies. Lineage markers are redistributed when a single marker fixes in the whole population. PCAs coloured for lineage markers, and composed per simulation on relative single-cell protein expressions, see “Methods” for details.

However, mutants with different metabolic repertoires continuously arise and compete for dominance within populations and all populations are highly diverse throughout the evolutionary simulations. As only very few genotypically identical individuals are present at any given time, we found it useful for interpretation and visualisation purposes to classify microbes based on their “metabolic genotype”: a binary representation that indicates the presence or absence for each of the 59 metabolic genes and transporters in the genome. Tracking the abundances of these metabolic genotypes over time shows that the evolutionary dynamics are complex and characterised by clonal interference and frequent hitchhiking, leapfrogging and horizontal gene transfer (see Muller plots in Fig. 2).

To condense these complex dynamics, we use lineage tracking and analyse the cell proteomes of these lineages. This reveals that some of these heterogeneous communities are dominated by a single lineage that performs all the metabolic functions outlined above by itself (see Fig. 2a–d; Supplementary Fig. 1 for lineage markers for all 60 populations), while other communities diversify in two complimentary, cross-feeding lineages that specialise in producing one, and importing the other building block (Fig. 2e–h). These cross-feeding lineages form interleaved patterns in the spatial environment, and quickly mix when separated from each other (see Supplementary Fig. 6 and and Supplementary Movie 1). In some communities, cross-feeding or autonomous epochs that last tens of thousands of generations change by quickly switching strategy (Fig. 2i–l, from here on we refer to “autonomous”, “cross-feeding” and “switching” community types). Switching occurs only occasionally, and typically once either strategy is established in a community it lasts until the end of the simulation. So, even though new mutations continue to fix in the population and metabolic networks remain in flux, the metabolic strategy of a community is very stable.

**Fig. 3:**
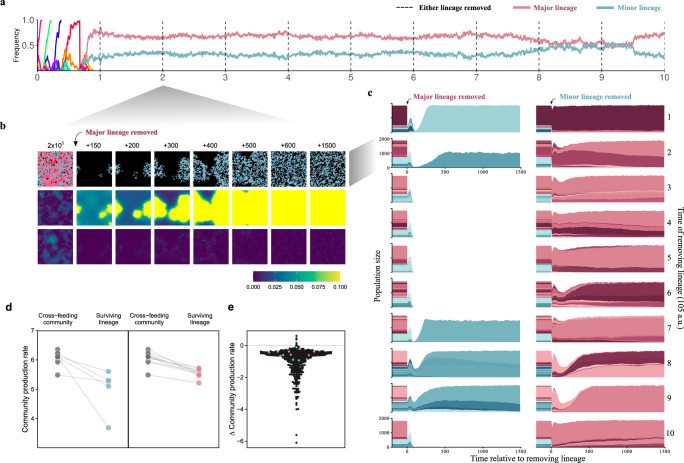
**Metabolic dependencies in cross-feeding communities**. We tested metabolic dependencies in 29 selected populations by removing either lineage at different time points after cross-feeding evolved, and without allowing further mutations to occur. **a**–**d** Example of 2 × 10 tests of metabolic dependency in replicate population 23. Times indicated with dashed lines in **a**. **b**. When removing the major lineage (pink) at *t* = 2 × 10^5^, most microbes of the remaining lineage (blue) die out. However, a rare mutant is able to grow by itself, though it cannot import building block 1 and does not reach a high abundance. **c** Outcome of removing lineages for all time points in (**a**), with different metabolic genotypes within each lineage indicated with shades of the lineage colour. Typically, the minor lineage goes extinct or contains only few mutants that survive in isolation, reflecting obligate dependency on the major lineage. In contrast, microbes in the major lineage can mostly survive without the minor lineage. These dependencies are not constant over evolutionary time as metabolic genotypes that dominate within each lineage change. Note that directly following lineage removal, all remaining lineages can initially quickly grow on the limited store of building blocks that were produced by partner lineage and are still present in the environment. **d** Community production rates before and 1500 time steps after lineage removal. All surviving minor and major lineages have higher growth rates in the context of the original cross-feeding population. **e** Difference in community growth rate for surviving lineages in 484 tests of metabolic dependency in 29 populations. In total, 407 out of 412 (98.8%) tested cases that survive removal have reduced growth rates in isolation. Surviving lineages shown in **a**–**d** are highlighted.

To investigate the nature of the cross-feeding interaction, we examined whether lineages could survive in absence of the other. Specifically, at different time points after the two lineages emerged, we tested metabolic dependencies in the standing diversity of cross-feeding populations by removing all microbes from one lineage and preventing further mutations to occur in the remaining lineage (Fig. 3a–c). We find that cross-feeding communities generally consist of a major lineage that produces both building blocks and can survive by itself, and a minor lineage that is obligately dependent on the major lineage for one of the building blocks and goes extinct when the major lineage is removed, barring a few rare mutants (Fig. 3c). These dependencies are not constant during the simulation, but can increase, decrease, completely switch direction and change to fully co-dependent, as reflected by large changes in size of the population bottleneck following lineage removal (Fig. 3c) and changes in the ratio that cross-feeding lineages occur in a community during the main experiment (Fig. 2; Supplementary Fig. 1). However, both major and minor lineage nearly always grow faster in the presence of their partner (98,8% of cases that survive, see Fig. 3d, e; Supplementary Fig. 2), supplementing their own metabolism with building blocks produced by the other lineage.

At any given time during a simulation mutants with the opposing strategy can be found within a community, but even though simulations last tens of thousands of generations communities only switch strategy a couple of times and most communities (45/60) do not switch at all (Fig. 2; Supplementary Fig. 1). For example, in cross-feeding populations autonomous mutants can easily evolve via horizontal gene transfer between lineages with complementary metabolic networks. When a cross-feeding lineage is removed, such mutants in the remaining lineage can successfully take over and found a new, completely autonomous population, but their growth rates are higher in the context of the original cross-feeding population where they exploit the environment created by the whole ecosystem (Fig. 3d, e; Supplementary Fig. 3). This explains why they cannot replace the resident cross-feeding community in the main evolutionary experiment, where they sometimes reach substantial fractions but are typically only transiently present. Similarly, in autonomous communities gene loss can produce mutants that specialise on producing or importing only one building block, but these fail to invade in the resident population that imports and produces both building blocks. Apparently, both cross-feeding and metabolic autonomy are eco-evolutionary attractors that are stable against invading mutants of the opposed strategy, with only occasional occurrences of populations switching between them. Since all simulations started from the same ancestral community, this shows evolutionary contingency determines what kind of community evolves. Before we further consider the consequences for predicting evolution, we first need to understand exactly what determines which strategy evolves.

### The evolution of cross-feeding is not explained by protein cost

The Black Queen Hypothesis explains the evolution of cross-feeding through the adaptive loss of costly biosynthetic genes for metabolites that are produced by community members and publicly available^27–29^. In our simulations, the evolution of cross-feeding is characterised by loss of genes for building block synthesis and/or transporters, and results in smaller genomes for cross-feeding compared with autonomous strategies. As we assume an explicit cost for protein expression and essential building blocks are an “inescapable public good” because they are released into the environment when cells die, evolution of cross-feeding could thus be driven by Black Queen dynamics.

To test this we study the effect of varying the cost for protein expression on the evolution of metabolic strategies. Surprisingly, the emergence of autonomous, cross-feeding and occasionally switching communities is robust to increasing or decreasing the costs of proteins expression an order of magnitude (Supplementary Fig. 4). Although some of the dynamics change (for example, lower expression costs allow larger genomes to evolve and increased expression costs cause the evolutionary dynamics to slow down), both strategies evolve under all conditions and are stable eco-evolutionary attractors. Thus, even though in our model the production of building blocks acts as a public good and protein expression has an explicit cost that can be reduced by gene loss, the evolution of cross-feeding is not driven by gene loss to escape this cost.

### Trade-offs emerge during the evolution of metabolic networks

**Fig. 4:**
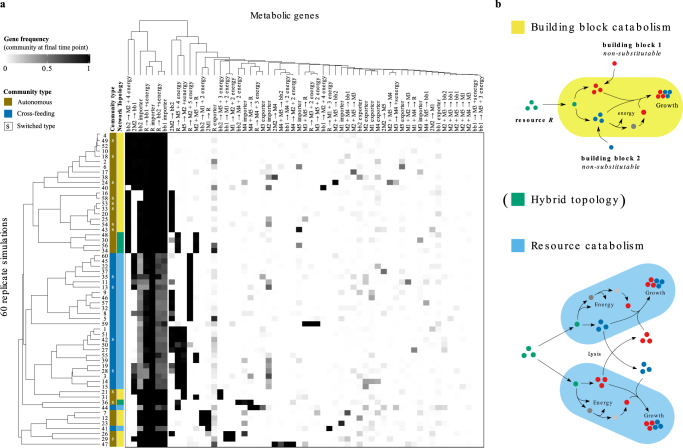
**Emergent metabolic strategies differ in their energy metabolism**. **a** Heatmap showing the frequency of 59 metabolic genes (columns) in 60 evolved communities (rows) at the end of the simulation. Cross-feeding communities (dark blue label) and single-lineage autonomous communities (in mustard) cluster mostly together, but no single gene is associated with either metabolic strategy. Instead, the topology of the evolved metabolic network determines community strategy. **b** Examples of metabolic networks with different topologies. Topology is determined by the substrate of the energy reaction (resource or building block), and networks with the same topology may differ in the specific reaction used to produce energy and other reactions. All communities that degrade resource *R* for energy follow the cross-feeding strategy (light blue), while in contrast all autonomous communities degrade building block *B*_1_ or *B*_2_ for energy (yellow). 15 out of 60 communities switched strategy during the evolutionary simulation (marked with letter "S" in **a**) in most cases because a mutant with the opposing network topology invaded and replaced the resident population. Some communities are formed by microbes with hybrid metabolic networks that degrade both resource and building block for energy (indicated in green) and can switch strategy without changing network topology.

To look for signatures for cross-feeding and autonomous strategies, we further investigated the diversity of metabolic networks that evolved. First, we clustered the final evolved populations at the end of the simulation based on metabolic gene frequencies in each population (see Fig. 4a). This shows that all populations share a core set of five genes for the uptake of the food resource and production and uptake of both building blocks. In cross-feeding communities, the genes for production and uptake of building blocks are only present in subsets of the population, reflecting how these communities have a distributed metabolic network. Note that populations strongly differ in which reaction is recruited to produce energy, and how byproducts from this reaction are further metabolised. Typically, a single dedicated energy reaction fixes in a population. Although clustering is dominated by individual energy generating reactions which clusters autonomous and cross-feeding populations with a few exceptions (Fig. 4a), no single gene acts as a signature for either community type.

**Fig. 5:**
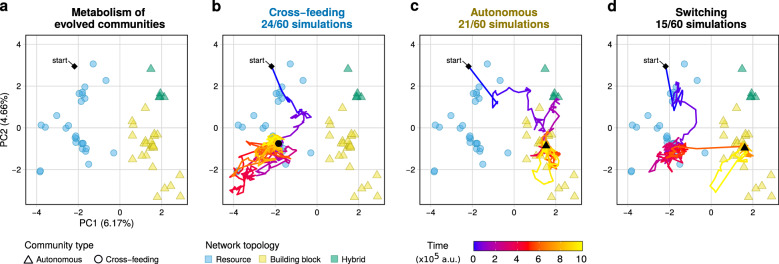
**Evolutionary trajectories towards community attractors**. **a** PCA of gene frequencies of 59 metabolic genes in 58 communities over the whole duration of the experiment. One dot represents one community. For clarity, only the initial community and final time point of the simulations are shown. This separates communities by strategy along the first component, and reveals that topology of the evolved metabolic network determines metabolic strategy of the community: networks with reactions that degrade resource *R* for energy cross-feed on building blocks, whereas networks with reactions that degrade building block *B*_1_ or *B*_2_ for energy remain metabolically autonomous and consume all building blocks from the environment. **b**–**d** Evolutionary trajectory showing all time points in the PCA for a community that **b **evolves cross-feeding (community 9), **c** metabolic autonomy (community 4) and **d** switches between strategies (community 18). For visualisation purposes outlier populations 59 and 47 were omitted from this analysis (see Supplementary Fig. 5 for analysis including these outliers).

**Fig. 6:**
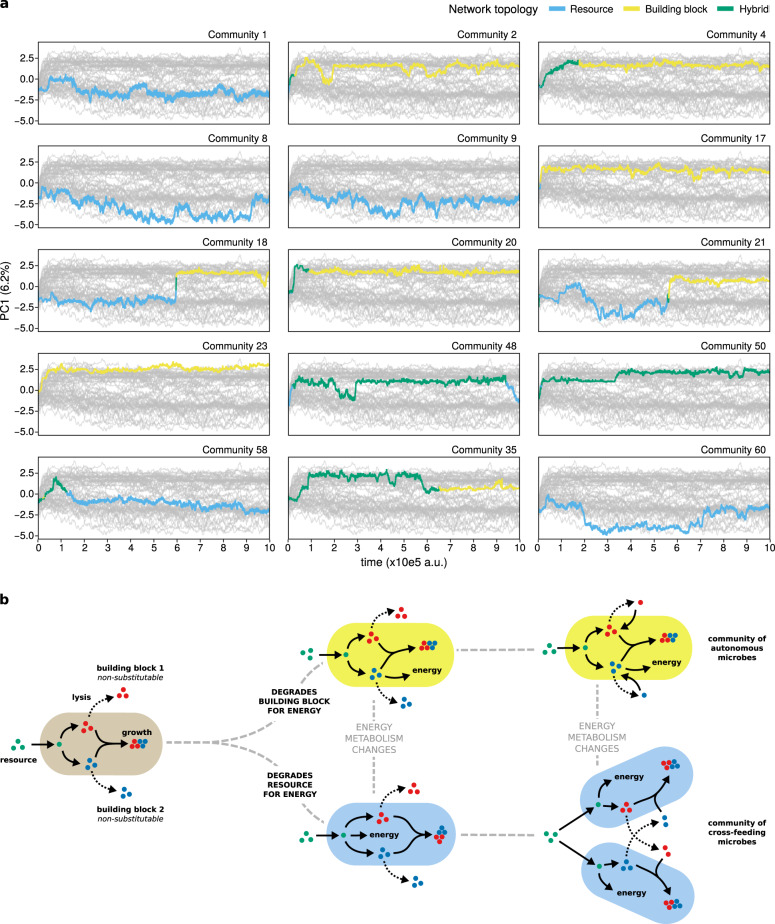
**Prior metabolic adaptations constrain future ecological roles**. **a** Evolutionary trajectories of example communities (first component from PCA in Fig. 5 v.s. time) towards cross-feeding (negative *y*-value) or autonomous (positive *y*-value) strategy, coloured for topology of the metabolic network. Grey lines indicate trajectories of all other communities. Changes in dominant network topology cause a switch in community strategy. **b** Cartoon of evolutionary trajectories. Earlier metabolic adaptations that fix in the initial population dictate final eco-evolutionary attractor, but are an evolutionary contingency. However, prediction is limited because the duration of each depicted stage is unpredictable, and cases where mutants with an alternate network topology invade and replace the population (dashed arrow in **b**, population 18 and 21 in **a**) are possible.

Next, as metabolic strategies are stable during long-term evolution even though metabolic networks continuously evolve, we consider how these gene frequencies change over the complete duration of the simulation by PCA (Fig. 5; Supplementary Fig. 5). We find that the major component separates cross-feeding and autonomous communities. Moreover, based on the energy reactions recruited by each strategy, the metabolic networks can be classified in two different topologies that associate exclusively with either strategy: networks that degrade the food metabolite for energy (i.e. R  →  energy + byproduct) are found in cross-feeding communities, and networks that degrade a building block for energy (i.e. B1 or B2  →  energy + byproduct) in autonomous communities (Fig. 4b). Further support that links network topology to community strategy comes from communities that switched between strategies. Here, a switch from cross-feeding to autonomous (or vice versa) is accompanied by a simultaneous switch in network topology, and when a switch occurs communities move along the first principal component accordingly (Figs. 5d and 6a). Finally, communities can be composed of microbes with hybrid metabolic networks that degrade building blocks as well as food for energy (Figs. 4, 6). Interestingly, within a hybrid metabolic network one type of energy reaction appears dominant, as communities with such networks follow either a cross-feeding or autonomous strategy and do not mix different strategies within a community. Such communities can also switch strategy (and move accordingly in the PCA) without changing their network topology.

Why do these topologies determine metabolic strategies? The amount of energy available to a microbe is limited, and as a consequence, importing more of one metabolite trades off with importing others, depending on what metabolite is used as an energy precursor. If microbes create energy by degrading the food resource, taking up other metabolites such as building blocks lowers the cell’s energy budget. The amount of additional metabolites that can be imported is thus constrained for metabolic networks with this topology. As building blocks are produced and accumulate in the environment, this creates two niches (i.e. one for each building block) that can be exploited by different lineages. In contrast, when building blocks are degraded for energy, importing them increases the energy budget and does not trade-off with importing the food resource, allowing individual microbes to import both types of building blocks as well as retain their competitive ability for the food resource. Furthermore, as only one of the two building blocks is used for energy in autonomous communities, a cross-feeding scenario with this topology would be inherently asymmetrical and unstable, as one lineage would be dependent on the other for both energy and building blocks, while the other lineage only requires the complementary building block.

### The evolution of cross-feeding requires spatial structure

Recent experimental and theoretical work^11,48–51^ re-emphasised the importance of spatial structure and local interactions on eco-evolutionary dynamics, and metabolic division of labour in particular. In our evolutionary experiment microbes reshape the composition of the local environment through metabolic activity, and cross-feeding lineages self-organise into interleaved spatial patterns, locally enriching it for one of both building blocks (see Figs. 2, 3b; Supplementary Movie 1 and Supplementary Fig. 6). To test whether spatial structure was necessary for cross-feeding to evolve, we re-ran the experiment 18 times starting from the same ancestral population while simulating well-mixed but otherwise identical conditions. No cross-feeding lineages emerged, even though metabolic networks evolved that reliably associated with cross-feeding strategies in unmixed conditions. Moreover, when we stopped mixing, populations with the cross-feeding-associated topology quickly diversified in two cross-feeding lineages, while communities with an autonomous-associated topology remained metabolically autonomous, signifying that it is the interplay between environmental structure and evolved metabolic constraints that drives cross-feeding.

Finally, reasoning that long-term coexistence might result in increased robustness of the cross-feeding interaction, we tested the ecological and evolutionary stability of cross-feeding communities from the original experiment by transfer to a well-mixed medium. Specifically, we subjected seven randomly chosen cross-feeding populations to well-mixed conditions at varying time steps after cross-feeding evolved, while either allowing or preventing further mutations to occur. In all “ecology-only” tests (i.e. without mutation), cross-feeding is stably maintained, and population size and community productivity increase. The increased productivity makes intuitive sense, as mutants that are less productive are outcompeted and cannot re-appear due to lack of mutations. Moreover, under unmixed conditions, local reproduction and metabolite diffusion limit the interface between both lineages and therefore reduce efficient exchange of building blocks. In contrast, when mutations are allowed under mixed conditions, all cross-feeding communities are quickly taken over by autonomous mutants. Strikingly, the resulting autonomous communities have smaller population sizes and productivity than their ancestral cross-feeding community. This shows that while spatial structure puts an upper limit to the efficiency of cross-feeding, it also protects against autonomous metabolic strategies. Consistent with previous results^32,51^, we find that spatial structure is needed to evolve and maintain metabolic cross-feeding and also find that whether cross-feeding evolves or not depends on constraints of previous metabolic adaptations. As microbes evolve to produce more energy from either the resource or one of the building blocks, importing one metabolite trades of with importing others. We find that the shape of this trade-off is an evolved property of the metabolic networks and the local environmental niches they construct.

### Metabolic strategies are an evolutionary contingency

Our results demonstrate that the topology of the evolved metabolic network, combined with spatial structure, determines whether cross-feeding evolves or not. Which topology evolves in a population is arbitrary and often establishes early on. For simulations where the cost of protein expression is increased, this topology often fixes up to tens of thousands of generations before metabolic networks “mature” by making use of all building blocks that accumulate in the environment. What eco-evolutionary strategy will evolve when microbes finally evolve to tap into that source can be predicted from the evolved topology (see Fig. 6b), realising a fate already cemented earlier in its evolutionary history. However, it is interesting to note that exact prediction is limited by several factors. Firstly, evolution of the topology of the metabolic network is typically “founder controlled”, where the energy reaction that establishes itself first in the community quickly accumulates more beneficial mutations and is never outcompeted by other energy reaction genes that are discovered later on. However, mutants with a different energy type occasionally do invade and replace the original population, changing community fate (Fig. 6a). Secondly, microbes that have a hybrid metabolism can switch between strategies as they evolve and different energy reactions dominate the metabolic network or are lost (Fig. 6a).

Concluding, a combination of contingency and predictability is manifest in our eco-evolutionary modelling experiment. Given the topology of the metabolic network that evolves, in the long run, and with intermittent metastable states, the type of community which evolves is predictable.

## Discussion

Previous experimental studies have shown repeated evolution of cross-feeding^8,13,14,43,52^, and the emergence of cross-feeding is typically interpreted as driven by physiological constraints. By explicitly incorporating constraints such as trade-offs in resource uptake or production^25^, or a limit in the maximum number of reactions within a single organism^26^, theoretical approaches demonstrate how these constraints can force metabolic specialisation and drive the evolution of cross-feeding. Here, rather than pre-defining such physiological constraints in our model, we demonstrate how metabolic constraints can themselves evolve, and whether or not they are driving cross-feeding is a historic contingency.

Next to metabolic constraints, other factors important for the evolution of metabolic exchange have been previously identified. For example, in batch culture repeated cycles of feast and starvation generate temporal niches that explain stable coexistence of cross-feeding ecotypes^7,8,25,37,38^. In these studies, cross-feeding is unidirectional, i.e. one lineage feeds on metabolic byproducts of the other. In contrast, we here show bidirectional cross-feeding of two non-substitutable building blocks, and in a constant environment. This matches recent experimental observations of the evolution of cross-feeding on essential nutrients^43^, and stable coexistence mediated by uptake of debris from lysed cells of a coexisting lineage^8^.

Another suggested driver for the evolution of cross-feeding is that the collective exploitation of resources can be more efficient or productive than individual-based solutions^19,26,53,54^. In our simulations, autonomous mutants in cross-feeding communities are indeed less productive than the resident cross-feeders (Fig. 3; Supplementary Figs. 2, 3). However, communities that evolve a completely autonomous strategy are equally or more productive than cross-feeding communities, showing that productivity alone may not be sufficient to explain widespread metabolic-dependencies observed in nature. Thus, we should realise that community productivity itself is an evolved property of the evolved metabolism, and that division of labour is not necessarily more productive, as productivity depends on evolved metabolic constraints.

Metabolic dependencies can also arise due to non-adaptive gene loss caused by genetic drift in small populations^55^. In our model this seems not to be the case as cross-feeding has a clear adaptive benefit (Supplementary Fig. 2), and lost functions are easily regained by HGT.

Recent experimental work showed that initially clonal microbial communities quickly become genetically heterogeneous^9,44^, and that in the resulting complex evolutionary dynamics the fate of individual mutations does not only depend on that specific mutation’s fitness, but mainly on what new mutations accumulate in that background^44^. Likewise, we find that when no further mutations are allowed to occur, autonomous mutants already present in cross-feeding population never fixate in the well-mixed selection regime, but when they can acquire more mutations always take over and dominate evolutionary outcome. In other words, the outcome of evolution (i.e. type of community that evolves) depends critically on interlocking evolutionary and ecological timescales, recapitulating earlier theoretical work^46,47^.

It is interesting to note that in each cross-feeding lineage more mutants gain partial dominance than is the case in autonomous lineages (Fig. 2). The amount of mutations in synthetic cross-feeding versus autonomous strains was recently studied in^56^. Contrasting with our results, they found that although, under strong selection, the sum of the mutations in the two cross-feeding strains was larger and more varied than in the autonomous strain, the separate cross-feeding strains acquired less mutations than the autonomous strain. We conclude that the speed and trajectory of evolutionary dynamics in relation to co-evolving, interdependent lineages under various selection regimes is a promising avenue of future computational, theoretical and experimental research.

For computational feasibility and simplicity, we here considered a simple metabolism with only two essential building blocks, while in reality many metabolites such as carbon and nitrogen sources, amino acids, nucleotides and vitamins are required for growth, and are also exchanged in microbial communities^23^. It would be interesting to extend the model metabolism with more building blocks and investigate whether the here reported distinct community types (metabolic autonomy and cross-feeding) are maintained, or whether communities with intermediate levels of metabolic division of labour evolve.

Interestingly, the dichotomy in community types we report here matches a recent paper that investigated the metabolism of natural communities composed of up to 40 species. The authors reported that natural communities in a wide range of environments are either highly cooperative, with smaller genomes and diverse metabolic dependencies, or highly competitive, and dominated by species with larger genomes and overlapping nutritional needs^57^. This suggests the result of our computational experiment is a general property of microbial communities. Furthermore, it would be interesting to investigate whether the rapid switching between cross-feeding and autonomous community types we find also occurs in natural communities. It is exciting to see that patterns emerging in the eco-evolutionary dynamics of our multi-level virtual microbes model are now detectable through metagenomic analysis of natural communities. We look forward to future cross-feeding of the two approaches to enhance our understanding of the microbial world.

## Methods

### Metabolic genotypes and the construction of Muller plots

We found it useful for interpretation and visualisation purposes to classify microbes based on their “metabolic genotype”: a binary representation that indicates presence or absence of each of the 59 metabolic genes and transporters in the genome, ignoring copy number variations, promoter strength and kinetic parameters. During simulations, we created a phylogeny with clades consisting of related microbes with the same metabolic genotype by tracking gain and loss mutations that changed this metabolic genotype and collecting abundances and ancestral relationships. For creating Muller plots, we subsampled the data every 500 time steps, and used a cut-off of 5% of maximum population size to remove clades that never became abundant. Plots were created in R with the GGMuller package^58^, which follows the convention to stack every new clade emerging from the centre of its parent clade in chronological order of appearance.

### Lineage tracking with renewing markers

To allow tracking of ecological and evolutionary dynamics, we used a technique similar to barcoding methods used in experimental evolution^59^. We label each microbe in the initial population with a unique neutral marker that it passes on to their offspring. If at any point during the simulation all living microbes have the same marker—indicating that they share a common ancestor that was alive when markers were distributed—markers are renewed by giving each microbe a new unique marker. Note that these markers only track lineage dynamics and not mutations, and lineages do not correspond directly to the clades of metabolic genotypes shown in the Muller plots. Markers do not reflect genetic or phenotypic identity nor provide high-resolution insight in the thousands of independent adaptive and non-adaptive mutations competing within a population.

### Diversity within replicate populations

To investigate different metabolic strategies of individual microbes within a community, we measured differential investments across all metabolic reactions by normalising protein concentrations of each metabolic protein to the total protein concentration in the cell. We collected protein data from all cells for time points (1, 2, ..., 10) ×10^5^ and then performed a separate PCA for each simulation.

### Testing metabolic dependency

To asses metabolic dependency of microbes in cross-feeding populations, we removed all microbes in one lineage (including internal metabolites) and set mutation rates to zero. Following removal, we continued the simulation for a maximum of 2000 time steps or population extinction. For 29 populations that evolved cross-feeding (all 21 cross-feeding populations and 8 randomly selected switching population), we tested both lineages at every multiple of 10^5^ time steps after cross-feeding evolved. In total, we performed 484 tests. We measured production rates of microbes at the time step just preceding lineage removal and, if populations survived, after 2000 time steps following removal.

### Diversity between replicate populations

We performed a single principal component analysis on the frequencies of all 59 metabolic genes in all 60 replicate populations over all time points in the main experiment. For clarity of visualisation, we omitted two outliers (population 47 and 59) from this analysis, which did not impact the result. See Supplementary Fig. 5 for an analysis including these outliers.

### Model description

VirtualMicrobes extends an earlier explicit model of genome evolution, metabolism and homoeostasis^60–62^ by adding a structured environment and emergent microbe-microbe interactions. It is a highly configurable framework designed to study emergent, eco-evolutionary microbial dynamics under a wide range of possible conditions. For clarity, here we only describe the configuration and features that are relevant to this study. A full manual is available at https://bitbucket.org/thocu/virtualmicrobes and https://virtualmicrobes.readthedocs.io, and parts have previously been described in an earlier study^38^.

### Overview

VirtualMicrobes is an agent-based model, where each individual microbe occupies a grid point on a 2D plane. Population dynamics (i.e. competition, reproduction and death) play out every time step. Within a time step, cellular processes (cellular growth, gene expression, metabolic reactions including uptake, excretion and diffusion across the cell membrane, metabolite and protein decay) and environmental metabolite dynamics (food influx, degradation and diffusion between grid points) are updated using ordinary differential equations (ODEs).

### Metabolic universe

The metabolic universe is an a priori defined set of all metabolites and possible reactions between them. We procedurally generated a universe consisting of nine metabolites (a single resource *R*, two designated building block molecules *B*_1_, *B*_2_, energy carrier *E*, and four intermediate metabolites *M*_1−5_) with their own toxicity, degradation and diffusion parameters, and 43 metabolic reactions (see Table 1). This provides evolution considerable degrees of freedom for forming metabolic networks to produce the required building blocks and energy from the available resource.

**Table 1:** Metabolic universe: Using VirtualMicrobes, we generated an artificial biochemistry comprised of 9 metabolites, 8 importers, 8 exporters and 43 conversion reactions, with two non-substitutable building blocks and a single energy molecule.

Metabolite	Class	Diffusion rate	Degradation rate	Toxicity	Mass	Influx rate
R	Resource	0.011	0.0003	0.077	9	0.002
B1	Building block	0.011	0.0100	0.058	8	0
B2	Building block	0.012	0.0100	0.103	8	0
E	Energy carrier	0.050	0.1000	0.065	1	0
M1	–	0.014	0.0006	0.105	6	0
M2	–	0.019	0.0003	0.080	4	0
M3	–	0.013	0.0006	0.079	7	0
M4	–	0.014	0.0014	0.158	6	0
M5	–	0.016	0.0008	0.047	5	0

Reactions				
R → B2 + E	B1 → M4 + 2 E	M1 → M2 + 2 E	M2 + M5 → B1	M3 + M5 → R
R → B1 + E	B1 → M5 + 3 E	M2 + M3 → B1	M2 + M5 → M1	M3 → M5 + 2 E
R → M1 + 3 E	B2 → M1 + 2 E	M2 + M3 → B2	2 M1 → R	M3 → M4 + E
R → M2 + 5 E	B2 → M2 + 4 E	M2 + M5 → M4	2 M2 → M1	M4 → M2 + 2 E
R → M4 + 3 E	B2 → M3 + E	M2 + M4 → M3	2 M2 → B1	M4 + M5 → B2
R → M5 + 4 E	B2 → M4 + 2 E	M2 + M5 → M3	2 M2 → B2	M4 + M5 → R
R → M3 + 2 E	B2 → M5 + 3 E	M2 + M4 → B2	2 M2 → M4	M4 + M5 → B1
B1 → M1 + 2 E	M1 + M4 → R	M2 + M5 → B2	2 M2 → M5	M5 → M2 + E
B1 → M2 + 4 E	M1 + M2 → M3	M2 + M5 → R		
Importers: one for each non-energy metabolite (8 total)				
Exporters: one for each non-energy metabolite (8 total)				

### Transport

For all metabolites, transporters exist that import or export the metabolite across the cell membrane. Transporters ${\mathcal{T}}$ catalyse the transport of substrate *S* over the cell membrane by consuming energy metabolite *E*. Transport rate *v* is then given by Michaelis–Menten kinetics:

1 $$v={v}_{{\max }_{{\mathcal{T}}}}\cdot {\mathcal{[T]}}\cdot \frac{[S]\cdot [E]}{([S]+{K}_{S})\cdot ([E]+{K}_{E})},$$

where *K*_*S*_, and *K*_*E*_ are the Michaelis–Menten constants for the substrate and energy molecule. Depending on the direction of transport (importing or exporting) [*S*] is either the external or internal concentration of the substrate. *K*_*S*_, *K*_*E*_ and ${v}_{\max {\mathcal{T}}}$ are all freely evolvable parameters.

### Metabolism

Metabolic enzymes catalyse reactions of the general form:

2 $${R}_{0}+{R}_{1}+\ldots \longrightarrow \, {P}_{0}+\ldots ,$$

converting reactant metabolites {*R*_*i*_, …} to products {*P*_*j*_, …}. The rate of catalysis *v* is calculated with Michaelis–Menten kinetics as follows:

3 $$v={v}_{{\max }_{{\mathcal{E}}}}\cdot {\mathcal{[E]}}\cdot \frac{{\prod }_{R\in {\mathcal{R}}}[R]}{{\prod }_{R\in {\mathcal{R}}}([R]+{K}_{R})},$$

where $[{\mathcal{E}}]$ is the concentration of the enzyme catalysing the reaction, ${\mathcal{R}}$ the set of all reactant metabolites and *K*_*R*_ and ${v}_{{\max }_{{\mathcal{E}}}}$ are evolvable kinetic parameters of enzyme ${\mathcal{E}}$.

### Degradation and dilution of molecules

Concentrations of all molecules (i.e. proteins, product and metabolites) are adjusted according to the change in cell volume d*Vol*/d*t*. In addition, all molecules degrade with a molecule-specific rate degradation.

### Biomass production budget

Microbes convert building blocks *B*_1_ and *B*_2_ to a production budget ${\mathcal{B}}$ with rate *production*, favouring homeostasis of building block concentrations with:

4 $$production=\frac{[{B}_{1}]\cdot [{B}_{2}]}{1+\left(| [{B}_{1}]-\frac{[{B}_{1}]+[{B}_{2}]}{2}| +| [{B}_{2}]-\frac{[{B}_{1}]+[{B}_{2}]}{2}| \right)}.$$

This budget is spent for increasing cell size *cost*_growth_ and producing proteins by gene expression *cost*_expr_. Note that to maintain sufficient selection pressure to select for adaptive mutations as building block synthesis rates of populations increase during evolutionary simulations, we scale the amount of budget spent with ${{\mathcal{B}}}_{{\mathrm{scaling}}}$ :

5 $${{\mathcal{B}}}_{{\mathrm{scaling}}}=\frac{{\mathcal{B}}}{{\mathcal{B}}+{{\mathcal{B}}}_{{\mathrm{pop}}}},$$

where ${{\mathcal{B}}}_{{\mathrm{pop}}}$ is the time averaged population production value. The concentration of ${\mathcal{B}}$ then changes with a rate:

6 $$\frac{{\mathrm{d}}{\mathcal{B}}}{{\mathrm{d}}t}=production-cost_{{\mathrm{growth}}}-cost_{{\mathrm{expr}}}-degradation-dilution.$$

When cells grow, ${\mathcal{B}}$ is consumed proportional to the rate of growth:

7 $$cost_{{\mathrm{growth}}}=Vol_{{\mathrm{growth}}}\cdot {{\mathcal{B}}}_{{\mathrm{scaling}}}\cdot {\mathcal{B}}.$$

Total consumption of product for gene expression is summed over all genes:

8 $$cost_{{\mathrm{expr}}}=\sum_{i = 1}^{{N}_{{\mathrm{genes}}}}P{r}_{i}\cdot {{\mathcal{B}}}_{{\mathrm{scaling}}}\cdot {\mathcal{B}}.$$

### Cell volume growth

We assume that if microbes grow without being able to divide due to lack of space, they approach a maximum cell size $Vo{l}_{\max }$ with rate *g*. We implement a cost for maintaining cell volume with a small shrinking rate *s*. Volume changes with a rate:

9 $$\frac{{\mathrm{d}}Vol}{{\mathrm{d}}t}=Vol_{{\mathrm{growth}}}-s\cdot Vol,$$

where *Vol*_growth_ is given by:

10 $$Vol_{{\mathrm{growth}}}=g\cdot Vol\cdot \frac{1-Vol}{Vol_{\max }}\cdot {{\mathcal{B}}}_{{\mathrm{scaling}}}.$$

### Protein expression

Protein concentrations ${\mathcal{P}}$ for any given gene are governed by the function:

11 $$\frac{{\mathrm{d}}{\mathcal{P}}}{{\mathrm{d}}t}={Pr} \cdot {{\mathcal{B}}}_{{\mathrm{scaling}}}-degradation-dilution,$$

where *Pr* is the per gene evolvable parameter promoter strength. VirtualMicrobes features explicit transcription factor proteins (TF) that can modify protein transcription. TFs were not relevant for this study and not recruited during evolutionary simulations and are here omitted for clarity. See the full documentation for a description of TFs.

### Toxicity and death

We model death as a stochastic process depending on an intrinsic death rate *r* = 0.03, which is potentially increased when internal metabolite concentrations reach a toxic threshold. This cumulative toxic effect *e*_tox_ is computed over all internal metabolites *M* and the current lifetime *τ* of a microbe as:

12 $${e}_{{\mathrm{tox}}}=\sum_{m\in M}\int_{t = 0}^{\tau }f(m,t){\mathrm{d}}t,$$

with the toxic effect function *f*(*m*, *t*) for the concentration of metabolite *m* at time *t* with metabolite-specific toxicity threshold *tox*_*m*_

13 $$f(m,t)=\max (0,\frac{[{m}_{t}]-tox_{m}}{tox_{m}}).$$

This toxic effect increases the death rate *d* of microbes starting at the intrinsic death rate *r:*

14 $$d=\frac{{e}_{{\mathrm{tox}}}}{s+{e}_{{\mathrm{tox}}}}\cdot (1-r)+r,$$

where *s* scales the toxic effect. Microbes that survive after an update cycle retain the toxic level they accumulated so far. Cells can also die from starvation. When they are unable to produce enough biomass product to maintain the slowly decaying volume of the cell and cell size drops below a predefined threshold size, cells are automatically marked for death.

### Reproduction

Microbes grow and reproduce on a 45 × 45 grid, competing for metabolites and available space. A microbe can reproduce if it meets the minimal division size and there is an empty grid site in any of its eight neighbouring sites. When multiple microbes are eligible for reproduction in the same empty site, reproduction chance depends on the microbe’s production value *P*, with a dynamically scaled chance for a “no reproduction” event occurring. Cell volume is divided equally between parent and offspring (molecule and protein concentrations remain constant), and the genome is copied with possible mutations. Toxic effects built up during the parent’s lifetime do not carry over to offspring.

### Genome and mutations

The genome is a linear organisation of genes coding for enzymes and transporters. When a microbe successfully reproduces it divides and the offspring inherits a copy of the parent’s genome, which can then be subject to various types of mutations. Stretches of genes can be duplicated, deleted, inverted or translocated to another position on the genome (Table 2). At the single gene level, all evolvable parameters can mutate individually (Table 2). Horizontal gene transfer can occur on every time step. Innovations are modelled as HGT from an external (off-grid) source by drawing a random reaction from the metabolic universe with random parameters, and can occur every time step.

**Table 2:** Types of mutations and their probabilities.

Mutations	Description	Probability
Duplication	A stretch of 1 or more genes is duplicated. Stretch length is geometrically distributed with *P* = 0.3	0.001
Deletion	A stretch of 1 or more genes is deleted	0.001
Inversion	A stretch of 1 or more genes is inverted in order	0.001
Translocation	A stretch of 1 or more genes is moved to a random location on the genome.	0.001
Gene discovery	Per time-step probability of discovering a new gene (randomly parameterised)	0.0001
Horizontal Gene Transfer	Per time-step probability of copying a gene from a cell in a neighbouring site	0.0005
Point mutation	Per gene per generation probability of modifying a single parameter of a gene	0.02
**Evolvable gene parameters**	**Gene types**	**Value range**
Promoter strength	Enzyme, transporter	[0.01, 8]
*K*_*S*_	Enzyme, transporter	[0.01, 8]
*K*_*E*_	Transporter	[0.01, 8]
*V*_max_	Enzyme, transporter	[0.01, 8]
Exporting	Transporter	[True, False]

### Initialisation and experimental setup

We constructed an initial population of 2025 “minimally viable” microbes by generating genomes containing one importer gene for the food resource and 5 random enzymes, with the constraint that the resulting metabolic network was at least capable of producing the necessary building blocks and energy from the provided resource. Genes were randomly parameterised and ordered on the genome. In total, 60 identical copies of this population were propagated in parallel for 1 × 10^6^ time steps (corresponding to roughly 4 × 10^5^ generations) under the exact same conditions, differing only in the mutation seed.

### Statistics and reproducibility

The experiments and analysis conducted in this paper can be reproduced with the publicly available code.

### Reporting summary

Further information on research design is available in the Nature Research Reporting Summary linked to this article.

## Supplementary information

Supplementary Information

Peer Review File

Supplementary Movie 1

Description of Additional Supplementary Files

Reporting Summary

## Data Availability

The data shown supporting this study is available for download from 10.5281/zenodo.3840463^63^, with exception of the data used for creating the Muller diagrams in Fig. 2, Supplementary Fig. 6 and Supplementary Movie 1, which are available from the corresponding author upon request.

## Code availability

Full documentation, source code, and installation instructions for the VirtualMicrobes Python module are publicly available at https://bitbucket.org/thocu/virtual-microbes. This study used VirtualMicrobes version 0.2.5, which is archived at 10.5281/zenodo.3843609^64^. Custom scripts used for the analysis and creation of figures in this paper are available at https://github.com/meijer-jeroen/contingent-evolution-2020 and archived at 10.5281/zenodo.3841930^65^.
