# Supplementary material for: Contingent evolution of alternative metabolic network topologies determines whether cross-feeding evolves
Source: Commun Biol. 2020 Jul 29;3:401. doi: 10.1038/s42003-020-1107-x (PMC7391776; doi:10.1038/s42003-020-1107-x)
Supplement: Supplementary file 4 — Description of Additional Supplementary Files [file 42003_2020_1107_MOESM4_ESM.pdf]

Description of Additional Supplementary Files

File Name: **Supplementary Movie 1**
Description: **Cross-feeding lineages self-organise into spatially interleaved communities.**

Supplementary Movie 1 is a detailed illustration of how cross-feeding lineages self-organise into spatially interleaved communities. The top rows are initialised with cells of one lineage, while the bottom rows are filled with cells from the other lineage (coloured yellow and blue respectively). On the left-hand side, cells are depicted as circles. The size of the circle indicates the cell volume, and the brightness indicates the budget production rate. The right-hand side of the figure indicates which building blocks are present in the local environment, using a gradient from yellow to blue. A green colour thus indicates the building blocks are approximately equally abundant.

By inspecting this movie we can detect the following details:
* Cells are growing fastest (are brighter) at the interface between the lineages
* The yellow type is more dependent on blue than vice versa, growing mainly at the interface between lineage, and barely surviving on their own.
* Blue cells expand into the empty space where yellow has failed to thrive, consuming the abundance of necessary building blocks left behind by yellow.
* In response, yellow cells can grow and divide again, eventually resulting in an interleaved pattern of cells.

Note that this movie is merely an illustrative example of how cross-feeding cells can intermix, and the details on the inter-dependencies may differ across evolved communities.
