# Supplementary material for: Contingent evolution of alternative metabolic network topologies determines whether cross-feeding evolves
Source: Commun Biol. 2020 Jul 29;3:401. doi: 10.1038/s42003-020-1107-x (PMC7391776; doi:10.1038/s42003-020-1107-x)
Supplement: Supplementary file 5 — Reporting Summary [file 42003_2020_1107_MOESM5_ESM.pdf]

nature research

Corresponding author(s): Jeroen Meijer

Last updated by author(s): 22 May 2020

Reporting Summary

Nature Research wishes to improve the reproducibility of the work that we publish. This form provides structure for consistency and transparency in reporting. For further information on Nature Research policies, see our Editorial Policies and the Editorial Policy Checklist.

Statistics

For all statistical analyses, confirm that the following items are present in the figure legend, table legend, main text, or Methods section.

n/a	Confirmed	
☐	☒	The exact sample size (n) for each experimental group/condition, given as a discrete number and unit of measurement
☒	☐	A statement on whether measurements were taken from distinct samples or whether the same sample was measured repeatedly
☒	☐	The statistical test(s) used AND whether they are one- or two-sided<br>*Only common tests should be described solely by name; describe more complex techniques in the Methods section.*
☒	☐	A description of all covariates tested
☒	☐	A description of any assumptions or corrections, such as tests of normality and adjustment for multiple comparisons
☒	☐	A full description of the statistical parameters including central tendency (e.g. means) or other basic estimates (e.g. regression coefficient) AND variation (e.g. standard deviation) or associated estimates of uncertainty (e.g. confidence intervals)
☒	☐	For null hypothesis testing, the test statistic (e.g. F, t, r) with confidence intervals, effect sizes, degrees of freedom and P value noted<br>*Give P values as exact values whenever suitable.*
☒	☐	For Bayesian analysis, information on the choice of priors and Markov chain Monte Carlo settings
☒	☐	For hierarchical and complex designs, identification of the appropriate level for tests and full reporting of outcomes
☒	☐	Estimates of effect sizes (e.g. Cohen's d, Pearson's r), indicating how they were calculated

Our web collection on statistics for biologists contains articles on many of the points above.

Software and code

Policy information about availability of computer code

Data collection	Custom Python code was used to run simulations (Virtual Microbes 0.2.5). The code is publicly available.
Data analysis	Custom R code was used to analyze the data, which is publicly available.

For manuscripts utilizing custom algorithms or software that are central to the research but not yet described in published literature, software must be made available to editors and reviewers. We strongly encourage code deposition in a community repository (e.g. GitHub). See the Nature Research guidelines for submitting code & software for further information.

Data

Policy information about availability of data

All manuscripts must include a data availability statement. This statement should provide the following information, where applicable:

- Accession codes, unique identifiers, or web links for publicly available datasets
- A list of figures that have associated raw data
- A description of any restrictions on data availability

Data for all figures will be made publicly available upon publication as detailed in the data availability statement in the paper.

Field-specific reporting

Please select the one below that is the best fit for your research. If you are not sure, read the appropriate sections before making your selection.

☐ Life sciences ☐ Behavioural & social sciences ☒ Ecological, evolutionary & environmental sciences

For a reference copy of the document with all sections, see nature.com/documents/nr-reporting-summary-flat.pdf

Ecological, evolutionary & environmental sciences study design

All studies must disclose on these points even when the disclosure is negative.

Study description	We performed a parallel evolution experiment with digital organisms using the VirtualMicrobes modeling framework. In total 60 replicate simulations are discussed in the main text.
Research sample	The digital microbial populations were created with Virtual Microbes 0.2.5. On run time, every 50 time steps data was stored for individual (e.g. genome composition, cell size, protein concentrations), population (e.g. cell counts, allele frequencies, mean death rate) and environmental (external metabolite concentrations) observables.
Sampling strategy	Not applicable within simulations as analysis was performed on the full generated dataset. The number of replicate simulations used was limited by computational feasibility.
Data collection	Not applicable for computational simulations.
Timing and spatial scale	Not applicable for computational simulations.
Data exclusions	No data was excluded from the analyses. For visual clarity some figures show a randomly chosen subset of the the data.
Reproducibility	Available code and configuration files allow full recreation of the presented dataset, analysis and figure generation
Randomization	Not applicable because no a priori categorization was made
Blinding	Blinding is not relevant as all simulations were done under identical conditions and analyzed using the same computational pipeline.

Did the study involve field work? ☐ Yes ☒ No

Reporting for specific materials, systems and methods

We require information from authors about some types of materials, experimental systems and methods used in many studies. Here, indicate whether each material, system or method listed is relevant to your study. If you are not sure if a list item applies to your research, read the appropriate section before selecting a response.

Materials & experimental systems

n/a	Involved in the study
☒	☐ Antibodies
☒	☐ Eukaryotic cell lines
☒	☐ Palaeontology and archaeology
☒	☐ Animals and other organisms
☒	☐ Human research participants
☒	☐ Clinical data
☒	☐ Dual use research of concern

Methods

n/a	Involved in the study
☒	☐ ChIP-seq
☒	☐ Flow cytometry
☒	☐ MRI-based neuroimaging
