# Supplementary material for: Contingent evolution of alternative metabolic network topologies determines whether cross-feeding evolves
Source: Commun Biol. 2020 Jul 29;3:401. doi: 10.1038/s42003-020-1107-x (PMC7391776; doi:10.1038/s42003-020-1107-x)
Supplement: Supplementary file 1 — Supplementary Information [file 42003_2020_1107_MOESM1_ESM.pdf]

Reviewers' comments:

Reviewer #1 (Remarks to the Author):

This is an interesting theoretical paper on the evolution of metabolic cross-feeding in microbial communities. The authors use an agent-based 'toy model' of metabolism and other cellular processes, and let population of cells self-organize their behavior around a set of basic rules for growth, death and reproduction. Costs for metabolic strategies are included in the sense of the basic resource requirements for carrying functions in genomes and the resulting growth rates that are attainable relative to the availability of those resources, but fitness or cost-benefit tradeoffs of given strategies in the context of others are not explicitly modeled. Metabolic strategies that arise in the simulated communities are thus apparently stable ecological attractors emerging from the basic rules for individual cells. The authors find that communities follow two general eco-evolutionary trajectories: one in which communities of fully autonomous cells recycle their own waste, and one in which complementary metabolic types co-exist and are obligately depend on each other for key building blocks. The authors conclude that whether autonomy or cross-feeding emerges is predictable from the nature of the energy metabolism chosen early on the experiments, thereby highlighting a role for contingency in evolution.

The topic and questions are timely and interesting, the experiments seem well designed, the analyses thorough and the conclusions justified in the context of the results that were obtained, and so overall, I am supportive of this paper. I do have a few questions and comments that could help improve the paper.

General comments:

One comment I had is that the paper could use a bit more discussion on how to translate these theoretical findings and observations to real-world observations. There are two aspects to this. One aspect has to do with the use of a toy model and distinguishing between those findings that are likely to be general (i.e. they would hold if details of the model were changed) and those which are particular to the details of this instantiation of the model. One example of this is the observation that stable cross-feeding emergence around the exchange of the two building blocks that cells have been prescribed to need. In this case it seems to me the _nature_ of the cross-feeding might be something that emerges directly from the particular rules that are chosen (mutual exchange of two building blocks when those two building blocks are required), while the _emergence_ of cross-feeding might be more general and take other forms for other model variations. Less intuitively clear is if cross-feeding would still be stable if there were more than two building blocks – perhaps the model would stochastically "jump" between bi-directional solutions involving exchange of two out of the three building blocks, or perhaps there would be a more complex form of co-existence among multiple types that emerges, or something else altogether. I'm not suggesting the authors must do the experiment just outlined, although it would certainly be interesting, but I do think some further discussion would help the reader understand the degree to which different aspects of the conclusions might translate to the real world. There are several additional examples along these lines in the detailed comments below.

A second aspect to this same comment has to do with the meaning of the concept 'contingency' and how we should think about it, which in my view is often subtle. For example, it might indeed be that the emergence of some metabolic strategy is contingent upon the presence of a certain evolutionary precursor (in this case the emergence of cross-feeding emerging from certain energetic strategies) and so the outcome can indeed look like it depends on a "frozen accident". However, it is entirely possible that certain metabolic strategies are inherently better adapted to certain environments in the

first place, making them more likely to colonize those environments and thus making the emergence of new metabolic strategies derived from those initial colonizers more likely in those environments. In other words, in this case, the degree to which one considers the outcome to be the result of an optimization process or a frozen accident depends on how a frame one takes. Clearly there are still constraints from the underlying metabolism shaping the evolutionary trajectory even in that case, but understanding the nature of those constraints can be challenging and I think we should be carefully asking ourselves the question in which sense is a particular outcome contingent? I think this is important because a) environments in the wild are typically more complex and variable than in this experiment and the communities they harbor do not depend on only one resource and b) there is mounting evidence that many of the most abundant microbes across many environments have complementary and highly intertwined metabolisms. Examples include the process of anaerobic methane oxidation, microbial communities in the euphotic zone across the world's tropical and sub-tropical oceans and the apparently mostly symbiotic members of the 'candidate phyla radiation' that occupy groundwaters around the world. Cross-feeding thus emerges from a very wide range of starting metabolic strategies and those groups involved in cross-feeding are often highly abundant, suggesting there may yet be room for conclusions of it being driven by an optimization process. Just some food for thought.

A second general comment I had is that i personally found the logical flow of the arguments sometimes difficult to follow. This is not a criticism of the logic itself, it reflects gaps in my own knowledge as much as anything, and as mentioned above I think the analyses are quite thorough and well-done and the conclusions seem reasonable. But some of the underlying motivations and conclusions could be unpacked a little bit more to make it easier to follow for general readers that may not have all the necessary disciplinary background to understand certain concepts, terminology etc. Most of the detailed comments below are related to this comment, and generally they should not be difficult to address.

Again, overall, I think this is an interesting paper and I am supportive of it.

Rogier Braakman

Detailed comments:

[Disclaimer: these comments were written down during the direct reading of the text and so give a sense of potential confusions that could arise for others as well. Some points are resolved later on in the text, but I nevertheless left in the comment because it could provide avenues for reordering and streamlining the narrative flow. Comments were written from the perspective of an "outside reader" as much as possible and all are offered in the spirit of trying to help the authors strengthen their paper. I don't include specific comments for the abstract as i suspect the authors may want to rewrite to some degree around general comments]

Lines 24-25: which experiments? In the previous sentences the authors summarized general observations, not studies. should there be a "studies have shown" in the previous sentence? as written this sequence is throwing me off...

Line 28: the term "one-niche environment" is confusing... in my understanding a niche refers to the embedding of an organism in the environment within which it exists. Almost by definition, organisms produce waste as part of their life & death processes, and so as soon as an organism is placed within an environment, new opportunities for other organisms arise, and this continues at least until the elemental cycles are closed and mass flows are balanced. This makes me unsure of how to interpret

the meaning of the term "one-niche environment" or whether this term is appropriate from an ecological perspective. I realize the following sentences delve into exactly this idea that organisms create new niches simply through their existence, but this observation seems to me expected rather than surprising and so the notion of a "one-niche environment" seems misleading as a premise... this is not at all to say that the understanding the dynamics of how such niches are constructed and how they interact is not interesting or worthwhile - it very much is! - but the authors may want to consider somewhat reframing the perspective here and in the abstract

Lines 42-45: The earlier comment about framing aside, this is a very interesting and important question

Lines 46-48: I'm not quite following what this means - could the authors either unpack this sentence or give some examples? If this is the central motivation it seems important that readers can easily understand what it means. Contingency in what sense?

Lines 55-56: i'm not quite understanding. does this mean the authors initialize the experiment with a random functional metabolism and repeat it many times? If so, perhaps say explicitly. It seems to me that by definition one has to "pick" a metabolic starting point so that cells can grow and this automatically introduces biases

Line 56: single mutations? This seems important because the "distance" between metabolic phenotypes could be large and it is not a priori clear that those gaps could be bridged through single mutations.

Line 59-60: and what the role about single vs multi-gene mutations? HGT is an important force in microbial evolution and can introduce multiple new genes at once, thereby helping cross otherwise non-viable valleys, and so whether you include or not also would seem to dictate dynamics

Line 62-63: I'm not sure what this sentence means, maybe be more explicit

Line 68: I had not realized from the introduction that the study was utilizing a toy model of biochemistry and metabolism (and other processes). I think this should be made more explicitly clear, as it impacts not only my comments in the introduction but also the setting of baseline expectations regarding the universalities of findings (an important point).

Line 75: this again seems misleading because, unless the starting microbes possess all metabolic processes (i.e. thousands of reactions), one has to start somewhere and this inherently introduces biases, even if the nature of those biases is unknown. This comment is also relevant in the context of the "toy model" vs "real world" comment above

Figure 1:
1. a small subset of 43 means what? 10 reactions? how small of a network do you think is still realistically representative of how metabolism works?
2. it is common that material and energy resource are the same (e.g. like cells using glucose for carbon and for energy) or that energy resources are not molecules but light (i.e. phototrophs). Do the choices made in this regard in this model affect the outcomes of the study?
3. “Upon replication… …nearby microbes” this affects earlier comments, authors might want to "foreshadow" that mutations are not the only genetic force, as it affects the reading of the introduction

Line 91: ah, it seems that breakdown of resources can create "energy molecules”, resolving (at least partially) a comment in Fig. 1.

Lines 92-93: is this a comment about the specificity of enzymes or about network properties? I.e. do individual proteins each cover a small subset of reactions or does the set of proteins in a given genome cover a small subset of proteins (i suspect the latter but got a little confused on first reading).

Lines 93-96: this is key and inklings hereof should perhaps be clearer at the outset. The introduction talks only of "mutations" in very general sense.

Lines 99-100: why? because they are closer to division already? It seems to me this could be a possibly key criterion, as specialist cells often tend to be small (see e.g. many endosymbionts, epibionts of the candidate phyla radiation, or streamlined microbes in the surface oceans), and so i wonder if this could end up favoring generalists over specialists. Perhaps this could be addressed by clarifying how cell size is modeled in general (can it vary, how is it bounded, etc)

Lines 105-107: This again addresses a key question i had upfront and so should perhaps be foreshadowed earlier

Line 113: typo - attractors (plural)

Line 114: see earlier comment on the concept of a “one-niche environment”

Line 119-121: not quite clear what this means. Do you mean the barcodes are only tracked in a statistical sense at the population level? Because it seems to me that by zooming on particular barcodes that take over the population one can gain insight? Or am i missing something?

Line 125: what does “investigate single-cell proteomes” mean? Investigate how?

Line 126: i'm not quite following this conclusion... i can see that when there are multiple colors in the spatial panels those colors overlap in the PCA plot, but i am not following what that means

Line 127: there is no figure 2c. In general there seems to be a mismatch between the text and the figure+legend. The latter have panels a and b with subpanels, the text talks about panels a-f

Lines 127-128 (“these communities consist of …”): this is another step in the logic that i am not quite following. can it be unpacked a bit? how do you know these lineages are closely related and not convergently evolved?

Line 128-129: see previous comment. the connection between statements about phylogeny and the patterns observed are not entirely clear. i am probably being slow in my understanding, but perhaps others could find confusion along similar lines and a bit more explanation could help

Line 130-131: the populations in the first half of the experiment in Fig. 2b (timepoints in snapshots 1, 2 and 3) also look like they are interleaved, so the difference is not totally clear in this regard. Maybe explain more... do you mean that each type covers the whole box?

Lines 132-133: at first glance this seems more clear, but after a closer look the populations in snapshots 2 and 4 also seem to display separation in the PCA plots. what is the difference to the later time points? maybe just explain a bit more to clarify

Figure 2:
1. perhaps add a line that indicates which time points in the simulations represent the spatial

snapshots. I found myself trying to first understand how the two panels are related and then to figure out which time point it was
2. What exactly does "principal component analysis of single-cell proteomics" mean? something regarding correlations among expression levels? An intuitive understanding is not immediately arising and a clearer explanation along the way (not just in the methods) could help the reader along

Line 135: cite figure panel (and perhaps even time point range in the context of earlier comment) [see also corresponding comment in Fig. 2]

Line 138: this seems like an example of an outcome that arise directly from the construction of the model, in this case that it defines two building blocks. So perhaps this could almost be expected, in that if cross-feeding was to evolve it would (almost necessarily) involve those two building blocks? do the authors care to comment? would you expect that if there were three building blocks defined there would be an expected number of cross-feedings (the exact statistical prediction is escaping me) that would emerge and would you expect those to be similarly stable? Perhaps this is a large expansion of the study and so better suited as future work, but at least some discussion around these ideas could help the reader along

Line 140: why do the results suggest an obligate interdependence? From what you have shown, and given the following analyses, it seems to me the results raised the question of whether the interdependency is obligate or facultative rather than necessarily suggesting the former (i.e. obligate dependency). Or are there hints already in the results, or perhaps intuitions based off how the model works that makes you draw this conclusion, in which case the following was motivated by confirming this intuition? Either way i think the authors should clarify or modify

Line 141: see the preceding two comments regarding the formulation for the logic leading into performing this analysis - here it seems you are asking whether cross-feeding is obligatory

Lines 147-148: this brings up a question that i am realizing was also not fully clear from the preceding: was the cross-feeding of building blocks typically associated with the cessation of production of the metabolites that are imported from the neighboring microbe? It seems to me this could have been recognized from the properties of the network itself, even before performing these experiments, and indeed doing so would have informed the question/hypothesis of whether the cross-feeding was obligatory.

Lines 148-149: this comment suggest that cessation in the production of imported metabolites was the norm. Why do the authors think this is the case? If there is no pre-described fitness cost of carrying genes/proteins why should metabolite production cease at all? I can understand how stochastic processes could perhaps lead to loss of genes, but in the absence of fitness costs it is not clear to me why this is largely driven to fixation. This seems to be interesting emergent feature of the model...

Line 149: why do rare self-sufficient mutants have lower growth rates? perhaps the authors want to say a bit more?

Lines 156-157: it is not quite clear to me the degree to which this is built into the model... do you require an overlap between biosynthetic pathways and energy metabolic pathways, and if so, why, or can there be cases where they are independent? This is again relevant to the general comment of the degree to which expectations of simulation outcomes might preexist in the model formulation

Figure 3:

1. “However, both lineage harbor other, rare metabolic types that either produce (yellow) or import (purple, orange) both building blocks” this is hard to see in the second spatial panel of fig. 3b. I can clearly see blue and green and some orange, but i am not able to distinguish any purple and yellow. Maybe zooming in or use different color scheme so the rate mutants stand out more clearly, or indeed both (zooming + altering color scheme).
2. “We conclude that lineages consist of obligate cross-feeders” given how you in fact carefully build up to this conclusion, i would suggest rephrasing in the first paragraph as a question rather than a (perhaps premature) conclusion. See earlier comment.

Line 162: typo - do [not] import?

Line 165: how does this discovery work? Through mutations in the 'resource' importer, through mutations in one of the by-product exporters, both, or something else altogether? What i'm getting at here is again the nature of the "rules" in the experiment and the degree to which these build in particular vs general outcomes.

Lines 176-178: what fraction of experiments led to autonomy vs co-dependency? how common was either strategy?

Line 187: typo – update -> uptake?

Line 187 (‘dedicated energy reactions’): i think it should be clearer earlier on how energy metabolism and biosynthesis are related: are they intertwined or separate or can they vary across these? This seems like important information about the model construction that informs baseline expectations about what might be expected to emerge and what is surprising

Figure 4:
1. there is a shift between a black and a dark grey population that i am not fully following... the legend only contains the black (P++I++) and not the dark grey
2. In panel b again there is the rise and fall of various shades of green that i am now starting to surmise must be variants of the basic green (P-+I+-) type - i think this should be clear. And why do you think greens go through turnovers but not blues in this panel?

Line 193: recruited how? See earlier comment

Line 197 (“generic metabolic functions described above”): which functions? described where? The paper has discussed many metabolic properties and functions so far, so it's not clear to me what this refers to. To make it easier to follow either state explicitly, or perhaps give some examples

Lines 197-198: you might consider writing these out explicitly. I'm guessing you mean 1) import of R, 2/3) R -> B1/B2 and 4/5 import of B1/B2, but for me it would be clarifying and offer a guide post to see them written out

Lines 198-199: and also, perhaps be explicit how this is clear from the figure. I.e. add citation here "(cluster on right of Fig. 5a)" or something like that. And maybe also add again a highlight within the figure itself. This all relates to the fact that there is a lot of detail in Fig. 5a and it might be helpful to add some visual guides, both in the text and in the figure.

Lines 197-199: also, looking more closely at the figure, the cluster on the right end of the figure actually seems to have 6, not 5 reactions, with an additional 2M2 -> 2B2 reaction also included. Why do you leave that one out in your discussion here?

Line 201: this is somewhat difficult to see in the figure... energy reactions alternate with other metabolic conversions or uptake reactions in the clustering "tree" at the top of the figure so it really requires carefully looking and zooming in to see if all dark blue blocks are indeed energy reactions. Perhaps you could highlight the energy reactions using a dedicated and distinct color so it is immediately obvious that the blue blocks do indeed (largely?) fall into the energy categories

Line 201: i just realized there is another colored bar on the left that indicates the energy reaction... but how that bar relates to the larger 2-D plot is not entirely clear and requires some sleuthing. Indeed, there is at least one energy reaction cluster (M5 -> M2 + E) that emerges in the 2-D plot that is not included in the bar on the left. Perhaps this should all be explained a bit more (and double checked), while perhaps also implementing the suggestion from before so that the relationship between the bar on the left and the main plot is both visually and textually clear

Line 202: there also appear to be some clusters not related to energy metabolism (i.e. the 2M1 -> R reaction or the M1 importer) that could be used as counter examples. Or do those counter examples prove the rule?

Line 204: did you look at productivity (i.e. total biomass or numbers of cells or the like)? I am not finding clear indicators along these lines in the figures. Or do you just mean that cells that take up extra building blocks take over the population? If you mean the latter i would just say that

Line 207: why is your first hypothesis (that all communities would eventually recruit both building block importers) not discussed in the context of this statement, which immediately follows the hypothesis and already seems to counter it? why include the hypothesis if you do not intend to discuss?

Line 209: why did you opt for PCA as the type of analysis to address this question? I mean, the figure does show a clear separation in the PCA analysis, but because it isn't immediately intuitive what the principle component axes are, it might help the reader follow your logic if you explain why you went about it this way and not another

Line 210: see previous comment... what is this major component? are the components to do with concentrations of compounds or something else?

Line 211: Can you explain this a bit more? This seems like a key observation. But also, is it always true? It is a bit hard to see from the figure, and by zooming in i noticed that the green&yellow reactions occur both in self-sufficient communities and in (at least one) cross-feeding community. "In general" it does appear to be true, but that might be relevant caveat, as in one could (in theory) zoom in on that one exception and understand why it forms an exception

Line 212 (“two classes”): what about the M5 -> M2 + E reaction, which seems to fall into a different category? How does it fit in to your conclusions here?

Lines 219-221: ah, this is really key in the context of an earlier comment about not addressing a hypothesis, which was rather confusing until reaching this point in the text

Figure 5:
1. what is the difference between 'main experiment' and 'prediction experiment'? i think it should be clear in the figure, or at least mention and point to the main text for details
2. “populations do not cluster…energy reaction” – this sentence seems unfinished. maybe add "... type

[but instead cluster according to] energy reaction[s]" or else reformulate?
3. “This splits community along the first component” - see comment in main text: what is this component, and more generally, what are the variables going into the PCA analysis?

Lines 226-228 (“firstly”): can you cite an observation in one of your figures to highlight this point? are these the purple waves in fig 4a?

Lines 228-230 (“secondly”): why do you think this is?

Lines 230-232 (“thirdly”): this would seem to be the exception noted in an earlier comment... identifying "population 28" is difficult from the population numbering though, as the fonts are small and closely overlapping. Perhaps you want to again highlight this one somehow to help the reader? Also, what do you mean by "exactly mirroring" and why does that seem to be key to the emergence of stable long-term cross-feeding (as opposed to some kind of drift pushing out one or the other)?

Line 235 (“in rare cases”): can you again cite an example of this in one of your figures? Otherwise it is not clear if you are just speculating or whether you indeed observed this happening

Figure 6:
1. “…invade and replace the population…” can you add a cite to an example of this dynamic in one of your other figures?

Line 245 (“metabolic strategy”): you mean that evolution of metabolic strategies is an emergent pnhenomena at the ecosystem level, correct? because of course you seed your experiment with a metabolic strategy, so that is not emergent. Might be helpful to clarify

Lines 245-246: by identical here you mean specifically identical in that they import both building blocks, correct? But the internal metabolisms could still be different if i understand the model correctly... and moreover only some subset of initial metabolisms evolved the ability of importing both building blocks, correct? It might be helpful to clarify these points (probably easily addressed), since you are in effect picking the boundary conditions for these later analyses and conclusions from among the larger set of experiments you performed... What about if you zoomed out to that full set, was it at all predictable which populations evolved the ability to import both building blocks? Does stable cross-feeding emerge only for mutual compatibility? I guess i am just trying to understand if the conclusions about predictability & contingency change if you were to look at a different scale

Line 256 (“physiological constraints”): such as? It might not be clear to readers what this means...

Line 260: "physiological constraints" are discussed a few times here but this remains somewhat vague... might be helpful to clarify a bit more what exactly you mean. In the context of discussing theoretical studies you mention resource uptake/production and numbers of reactions, is that what you mean? But more generally, what do such constraints actually point to from a biological perspective? how does a tradeoff in uptake or production work, or why do the number of reactions matter? Explaining more clearly could help the reader understand how constraints emerge in the model being considered here.

Line 266: this relates to an earlier point that you seemed to zoom in on those experiments where bi-directional cross-feeding emerged... did you not observe uni-directional cross-feeding at all?

Lines 284-286: This statement feels abrupt, making it unclear how you arrive at your conclusion, or even what exactly the conclusion is. Readers may not generally know that these phenomena are

features of cross-feeding. They may not even know what some of the terms (e.g. negative frequency dependent selection) mean. So, you might want to elaborate here to help the reader along - start by briefly saying these phenomena are considered hallmarks of cross-feeding, explain what some of the terms mean and at least provide some citations supporting the claim. Even better would be to explain why these are considered hallmarks of cross-feeding.

Line 286-287: in fact, i'm not quite understanding how what you describe is different from the black queen hypothesis. Cells cannot lose costly genes (e.g. biosynthesis genes) _before_ they gain ecological access to the related metabolic products, otherwise the lineage would not survive. So the order of events you describe in your experiment - the emergence of cross-feeding followed by loss of genes for autonomy - seems similar to how you describe operation of the black queen hypothesis. Indeed that paper discusses several examples that seem quite similar to what you describe here.

Lines 288-289: this seems to me consistent with the arguments of the black queen hypothesis

Line 290-291: maybe the statement that you also often observe evolution of autonomy is key for why you argue for differences to the black queen hypothesis? Although i thought that earlier you had argued that autonomy evolves due to contingencies arising from which energy reaction is used.

Line 299-300: how did it reveal this conclusion? again i think it would help the reader if you could elaborate a bit and show your thought process

Lines 319-320: but why do you think that cross-feeding emerges when cells use an energy reaction in which energy is directly derived from the resource but not when it is derived from other reactions?

Reviewer #2 (Remarks to the Author):

The manuscript "Evolutionary contingency drives microbial metabolism, community structure, and its predictability" by Jeroen Meijer and colleagues presents a theoretical study about community structure dynamics in synthetic toy microbes. Specifically, authors using their model demonstrated that microbial communities could reach two very different stable states, namely, first with metabolic self-sufficiency, and the other that should will only exist through the cross-feeding. Authors concluded that these two states could be predicted from metabolic constraints in energy metabolism. The study is technically sound. The authors present a plausible evolutionary scenario and it and explain it using their theoretical "toy model", while modelling approach is robust. However, the work is not supported by any real biological data, thus in its current form remains a theoretical exercise.

Response to reviewer #1:

First and foremost, thank you for reviewing our manuscript so thoroughly. It is a true pleasure to receive such detailed feedback, and it has been taken to heart. That being said, let us first apologise for the delay in our revision. A minor bug was found in our program, and the model and data needed to be carefully re-evaluated. Although the storyline in the manuscript has not changed substantially, the text has undergone many changes. Further, as you remarked, there was room to improve the logical flow of our arguments. Although we will not be able to address your line-by-line comments, we feel that your comments were nevertheless thoroughly addressed. We hope you agree that the current version of the manuscript is much more easy to digest.

We do however wish to respond to your general comments, which were very helpful. Firstly, you pointed out it would be interesting to consider what dynamics may emerge is our model had not considered merely two non-substitutable building blocks, but more. As you suggest, it is indeed an intriguing thought that perhaps communities would stochastically switch between alternative cross-feeding solutions, or perhaps something else entirely may happen. Although we do not currently know how our experiment will play out when using *e.g.* three building blocks, our new manuscript does include populations with the ability to switch between cross-feeding (CF) and autonomous (AU) strategies (referred to in the current manuscript as switchers, SW). In other words, this alternative outcome indeed shows the "jumping" between alternative outcomes that you refer to. Either way, we now address this assumption of two building blocks in our discussion, and refer to some exciting new research that shows how similar "polarization" of CF and AU are found in natural communities.

Secondly, we agree with your second point that what we call a "frozen accident" is context-dependent. Indeed, some of these communities may well be more pre-adapted to other niches, more / less robust to mutations, or more / less evolvable, *etc*. However, the advantage of a modelling approach such as ours is that, despite these possibilities, we can be sure that these different network topologies are the result of contingent evolution. To call them an "accident", is in that regard indeed merely a figure of speech. Although we do discuss the effects of mixing and the costs of protein expression more thoroughly in the revised manuscript, we do not currently know how our communities will respond to fundamentally new challenges. We agree that this is an exciting subject for future studies.

Once again, thank you for your thorough reading of our manuscript. Your review was very helpful.

<u>Response to reviewer #2:</u>

Thank you for reading our manuscript. We would like to address your concerns about our study not having any biological data to support it. Although we have not ourselves performed experiments to test our analysis (it is a long-term evolution experiment after all!), there are many biological data available that are aligned with our data, as well as data that do not align with our results. That being said, the current manuscript has improved in communicating how our results fit into what is known from both experiments and theory. By reading the revised discussion, it should be clear how, given the treasures of data that are already generated by experiments, bioinformatics, and metagenomics, our work is in fact supported by plenty of biological data.

REVIEWERS' COMMENTS:

Reviewer #1 (Remarks to the Author):

The authors have significantly improved what was already a nice manuscript. They have identified and corrected a key bug in their code, have added some nice new results and have also improved the logical flow of the arguments, making them easier to follow and evaluate.

I have only a few minor comments remaining, partly in response to the newly added results/discussion:

- i still think the concept of a "one-niche environment" (abstract and p.6 section title) is confusing and a bit misleading as a premise, since waste is an inevitable byproduct of life, which creates new opportunities. "one resource environment" might be a better alternative...

- i would encourage the authors to just write out 'autonomous', 'cross-feeding' and 'switching' rather than abbreviating. Easier to follow for readers to follow and the abbreviation doesn't save that much space

- on page 7 it say that 'in only rare cases' do communities switch between autonomy and cross-feeding, but then in Fig. 2C it says that 14/60 of communities undergo the switch, while Fig. 6B says that 15/60 (please check numbers...), suggesting it's actually fairly common. I'm not sure how to reconcile these statements, maybe good to clarify. This point that switching is rare also comes back in other places

- i found Fig. 3 a little difficult to follow... "lineage a/b" are always labeled with light/dark blue, but then panel d has different colors and moreover many more colors, suggesting more population heterogeneity than panel a. What is the y-axis on panel d? partly because of the color switch the "lineage a/b remains" labels are hard to interpret because it's not clear which are a and b, and how then the dynamic relates to removing of either lineage.

- top of p. 11 it says that "SW communities are composed of microbes with metabolic networks that have a hybrid topology" but i'm not finding this to be true when i look at Fig.4 - there are only 5 green "hybrid" leaves on the tree, and of those only one has a diamond label linked to switching

- typos: "proteoms" (missing e) and "single, lineage" (extra comma) middle of p. 6, "Fig. , no single...community type 4a" (missing figure number and/or accidental movement of figure number to end)

Best wishes,

Rogier Braakman

Reviewer #1 (Remarks to the Author):

The authors have significantly improved what was already a nice manuscript. They have identified and corrected a key bug in their code, have added some nice new results and have also improved the logical flow of the arguments, making them easier to follow and evaluate.

Reply: We thank the reviewer for his nice comments.

I have only a few minor comments remaining, partly in response to the newly added results/discussion:

- i still think the concept of a "one-niche environment" (abstract and p.6 section title) is confusing and a bit misleading as a premise, since waste is an inevitable byproduct of life, which creates new opportunities. "one resource environment" might be a better alternative…

Reply: Agreed. We changed the term to 'one resource environment' as suggested by the reviewer.

- i would encourage the authors to just write out 'autonomous', 'cross-feeding' and 'switching' rather than abbreviating. Easier to follow for readers to follow and the abbreviation doesn't save that much space

Reply: We agree with the reviewer and have removed all use of these abbreviations from the manuscript.

- on page 7 it say that 'in only rare cases' do communities switch between autonomy and cross-feeding, but then in Fig. 2C it says that 14/60 of communities undergo the switch, while Fig. 6B says that 15/60 (please check numbers...), suggesting it's actually fairly common. I'm not sure how to reconcile these statements, maybe good to clarify. This point that switching is rare also comes back in other places

Reply:
What we try to contrast is that at any given time during a simulation mutants with the opposing strategy can be found in a community, but even though simulations last tens of thousands of generations and metabolic networks within the communities continuously change over this duration, communities only switch strategy a couple of times and most communities (45/60) do not switch at all. We made the following changes to make this more explicit:

Page 6 old text:

> These lineages in CF populations coexist for tens of thousands of generations, typically until the end of the simulation, but in some simulations single or repeated switching between AU and CF strategies occurs (SW populations, see Fig. 2c).

Page 6 new text:

> In some communities, cross-feeding or autonomous epochs that last tens of thousands of generations change by quickly switching strategy (Fig. 2i-l, from here on we refer to "autonomous", "cross-feeding", and "switching"' community types). Switching occurs only occasionally, and typically once either strategy is established in a community it lasts until the end of the simulation. So, even though new mutations continue to fix in the population and metabolic networks remain in flux, the metabolic strategy of a community is very stable.

Page 7 old text:

> Mutants with different metabolic types continuously emerge in AU and CF populations, but only in rare cases cause switching between CF and AU strategy of the community (Fig. 2, Supplementary Material 1). For example, autonomous mutants that import and produce both building blocks (Fig. 3d, coloured in black) emerge in CF populations and can reach substantial fractions, but are only transiently present. When the other lineage is removed, these AU mutants can successfully take over and found a new, completely AU population, but their growth rates are higher in the context of the original cross-feeding population, where they exploit the environment created by the whole ecosystem (Fig. 3b-d, Supplementary Figure 3). This explains why they cannot replace the resident CF community in the main evolutionary experiment. Similarly, in AU simulations, gene loss produces mutants that specialise on producing or importing only one building block, but these are only transiently present and fail to invade in the AU population that imports and produces both building blocks. Apparently, AU and CF are stable eco-evolutionary attractors, with only rare occurrences of populations switching between them.

Page 7 new text:

> At any given time during a simulation, mutants with the opposing strategy can be found within a community, but even though simulations last tens of thousands of generations, communities only switch strategy a couple of times and most communities (45/60) do not switch at all (Fig. 2, Supplementary Fig. 1). For example, in cross-feeding populations autonomous mutants can easily evolve via horizontal gene transfer between lineages with complementary metabolic networks. When across-feeding lineage is removed, such mutants in the remaining lineage can successfully take over and found a population of autonomous microbes, but their growth rates are higher in the con-text of the original cross-feeding population, where they exploit the environment created by the whole ecosystem (Fig. 3d-e, Supplementary Fig. 3). This explains why they cannot replace the resident cross-feeding community in the main evolutionary experiment, where they sometimes reach substantial fractions but are typically only transiently present. Similarly, in autonomous communities gene loss can produce mutants that specialise on producing or importing only one building block, but these fail to invade in the resident population that imports and produces both building blocks. Apparently, both cross-feeding and metabolic autonomy are eco-evolutionary attractors that are stable against invading mutants of the opposed strategy, with only occasional occurrences of populations switching between them.

We agree that our use of the word ‘rare’ in this context is confusing and should be avoided, and removed it completely or used ‘occasional’.

- i found Fig. 3 a little difficult to follow... "lineage a/b" are always labeled with light/dark blue, but then panel d has different colors and moreover many more colors, suggesting more population heterogeneity than panel a. What is the y-axis on panel d? partly because of the color switch the "lineage a/b remains" labels are hard to interpret because it's not clear which are a and b, and how then the dynamic relates to removing of either lineage.

Reply:
The reviewer is right in highlighting a poor choice in visualisation on our part. To make clear that lineages (microbes marked with neutral markers) are very diverse and contain many subpopulations with different metabolic repertoires, we made the following changes to the manuscript:

- To clear up the visual confusion we changed the colours in figure 3d (changed to 3b in the new text) so that the metabolic genotypes within a lineage have different shades of the lineage colour they are part of.

- In addition, we now refer to these subpopulations as ‘metabolic genotypes’ to indicate it is a classification based on genome content, clarifying the distinction from lineage markers. The term ‘metabolic genotype’ is used in studies that use genome-scale metabolic modeling to indicate the exact same thing, so it makes sense to also use this term here.
Previously, this classification was only explained in the Methods section at the end of the manuscript, but we now realise it is necessary to include it in the main text. We added a small section in the results on page 6 and now start our description of the evolution of community metabolism with this classification.

- top of p. 11 it says that "SW communities are composed of microbes with metabolic networks that have a hybrid topology" but i'm not finding this to be true when i look at Fig.4 - there are only 5 green "hybrid" leaves on the tree, and of those only one has a diamond label linked to switching

Reply:
The full sentence states that switching communities are composed of microbes with hybrid topologies, **or** are rare cases where topology switched. In other words, communities that switch strategy (as indicated with a symbol) either also switched metabolic network topology (autonomous → cross-feeding or vice versa) when this happened, or had a hybrid topology that did not change even though strategy did change.
To better explain this point we changed the order and expanded our arguments in this paragraph, and also avoid use of the word ‘rare’:

Page 11 old text:

> Interestingly, SW communities are composed of microbes with metabolic networks that have a hybrid topology (i.e. they degrade building blocks as well as food for energy), or are rare cases of simulations where one topology invades and replaces the other (Fig. 4, Fig. 6).

Page 11 new text:

> Further support that links network topology to community strategy comes from communities that switched between strategies. Here, a switch from cross-feeding to autonomous (or vice versa) is accompanied by a simultaneous switch in network topology, and when a switch occurs communities move along the first principal component accordingly (Fig. 5d, Fig. 6a). Finally, communities can be composed of microbes with hybrid metabolic networks that degrade building blocks as well as food for energy (Fig. 4, 6). Interestingly,within a hybrid metabolic network one type of energy reaction appears dominant, as communities with such networks follow either a cross-feeding or autonomous strategy and do not to mix different strategies within a community. Such communities can also switch strategy (and move accordingly in the PCA) without changing their network topology.

We also clarify the distinction between our analysis of the network topology in the final evolved populations (heatmap in Fig. 4) and analysis of the evolution of network topologies over time (fig. 5 and 6) by small changes in the text and figure legends.

- typos: "proteoms" (missing e) and "single, lineage" (extra comma) middle of p. 6, "Fig. , no single...community type 4a" (missing figure number and/or accidental movement of figure number to end)

Reply: We corrected the errors.

In the Results section we give considerable attention to the effects of spatial structure on the evolution of cross-feeding, and felt this part of our story was not represented well in the figures of our main text. We incorporated snapshots of the spatial grid in figures 2 and 3, and removed the corresponding Supplementary Figure which now became redundant. In addition we have made small aesthetic changes to Figure 1 (showing the overview of the computational model) to reduce visual clutter.

Best wishes,

Jeroen Meijer
Bram van Dijk
Paulien Hogeweg
