# Supplementary material for: Contingent evolution of alternative metabolic network topologies determines whether cross-feeding evolves
Source: Commun Biol. 2020 Jul 29;3:401. doi: 10.1038/s42003-020-1107-x (PMC7391776; doi:10.1038/s42003-020-1107-x)
Supplement: Supplementary file 2 — Peer Review File [file 42003_2020_1107_MOESM2_ESM.pdf]

Supplementary Materials for:

Contingent evolution of alternative metabolic network topologies determines whether cross-feeding evolves

Contents

1 Supplementary Figures **2**
1.1 Supplementary Figure 1: Neutral lineage markers for all 60 populations 2
1.2 Supplementary Figure 2: Community productivity of cross-feeding lineages 4
1.3 Supplementary Figure 3: Production rates of autonomous mutants in cross-feeding communities 5
1.4 Supplementary Figure 4: Effect of gene expression costs 6
1.5 Supplementary Figure 5: PCA of evolutionary trajectories of metabolic networks . . 8
1.6 Supplementary Figure 6: Cross-feeding lineages self-organise into a spatially interleaved community 9

1 Supplementary Figures

1.1 Supplementary Figure 1: Neutral lineage markers for all 60 populations

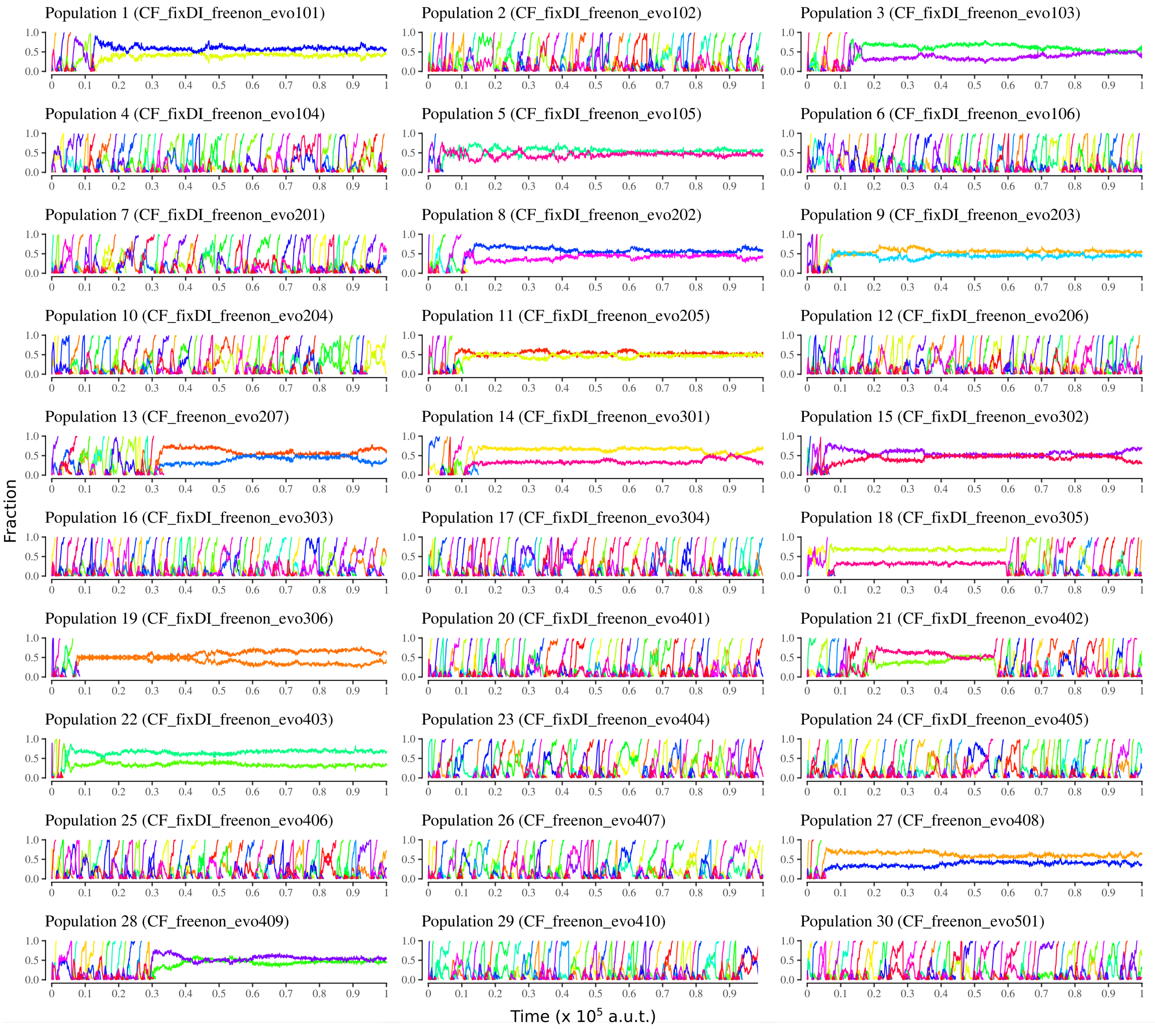

Figure 1: **Neutral lineage markers for all 60 populations of the main evolutionary experiment**. See main text for details.

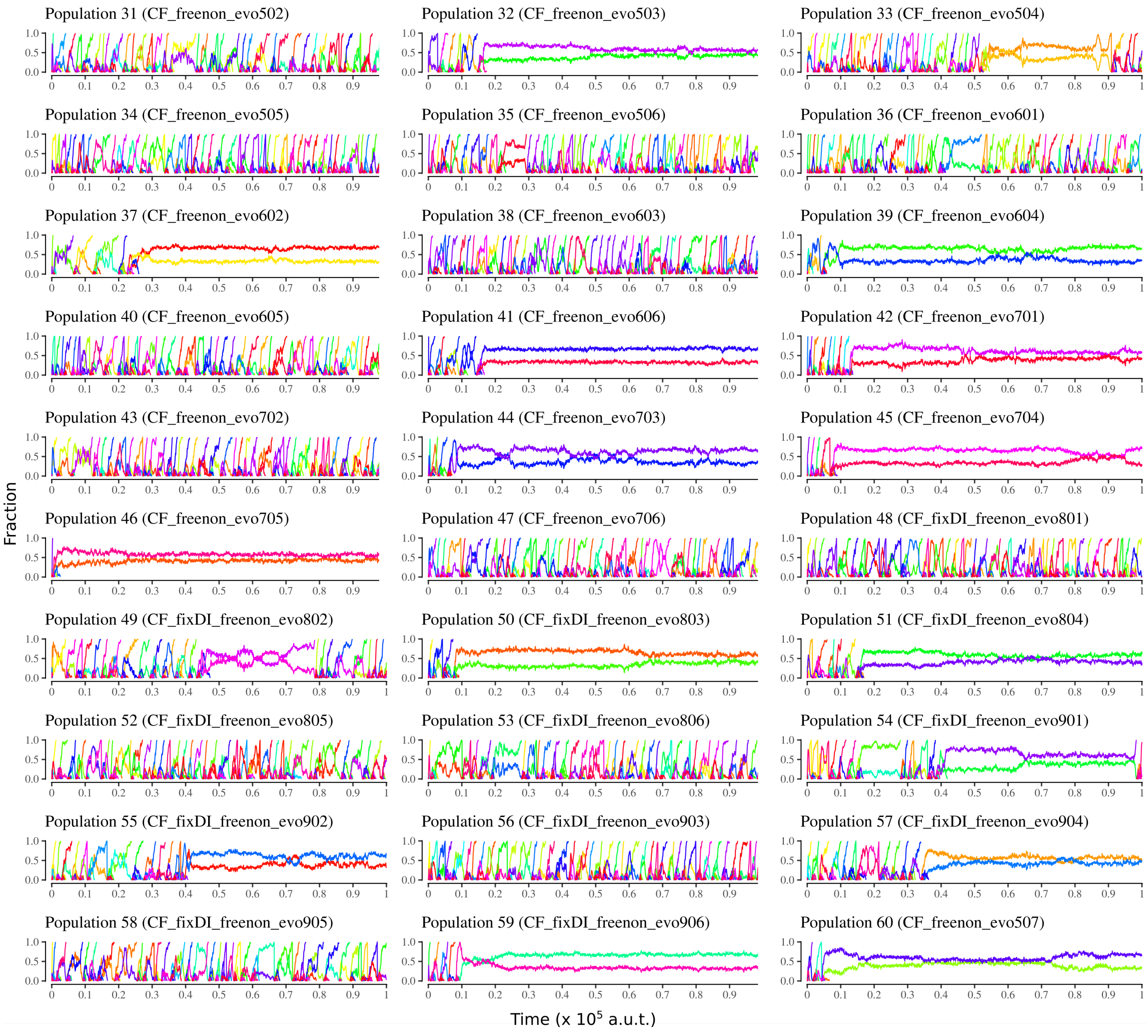

Figure 1: **(continued)**.

1.2 Supplementary Figure 2: Community productivity of cross-feeding lineages

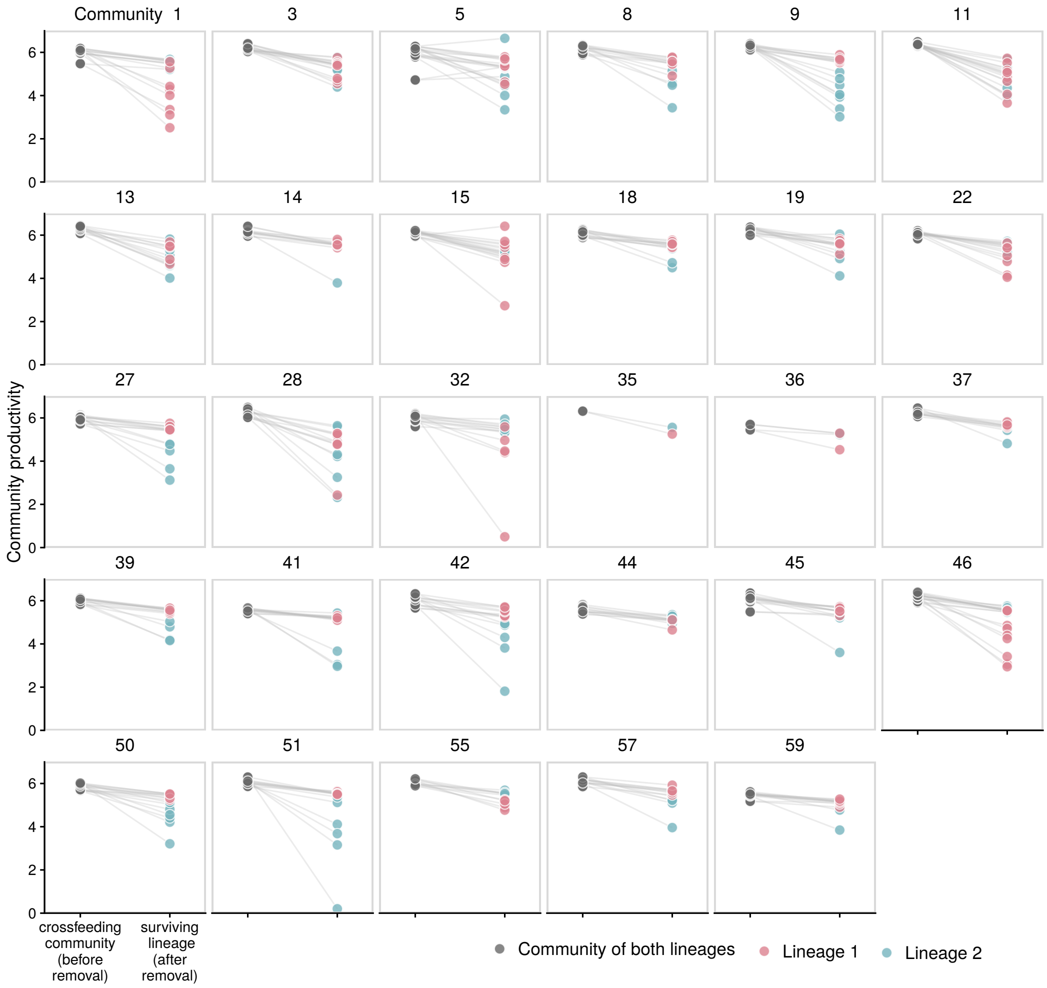

Figure 2: **Comparison of community production rate before and after removal of 1 cross-feeding lineage**.

1.3 Supplementary Figure 3: Production rates of autonomous mutants in cross-feeding communities

The autonomous communities in the main evolutionary experiment are formed by microbes that are individually capable of producing and importing both essential building blocks (B_1 and B_2). In contrast, microbes in the cross-feeding communities distribute these metabolic functions over the two lineages that are each specialised in producing one building block and importing the other. Autonomous mutants that import and produce both building blocks continuously evolve within these lineages (for an example see Supplementary Fig. 3a-b, indicated in black) and while these mutants sometimes reach a considerable frequency in the population, they only very occasionally invade and replace the resident community. By comparing the production rates of these mutants before and after removal of the partner lineage we can understand why this is the case. In the context of the original cross-feeding population these autonomous mutants have equal or higher production rates compared to the metabolic specialists that cross-feed (Supplementary Fig. 3), while their growth rates are much lower after the partner lineage is removed. This shows they depend on the environment of the cross-feeding population to maintain high growth rates.

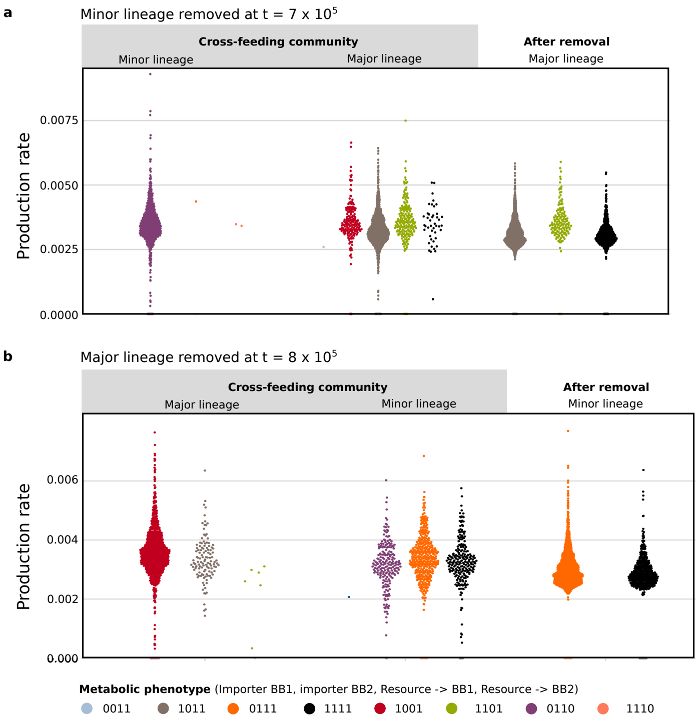

Figure 3: **Cellular production rates in a cross-feeding community**. Individual microbial production rates for two tests of metabolic dependency for population 45, shown in figure 3 of the main text. One dot represents one microbe. Colours indicate the presence or absence of four metabolic genes in the genome: importer for building block 1, importer for building block 2, resource $\rightarrow$ building block 1, resource $\rightarrow$ building block 2.

1.4 Supplementary Figure 4: Effect of gene expression costs

To investigate the effect of protein expression costs in our simulations we decreased and increased this cost tenfold (from 0.002 to 0.0002 and 0.02, respectively) and repeated the experiment for a total of 23 populations (Supplementary Fig. 4ab). Surprisingly, varying the cost of gene expression over two orders of magnitude again resulted in cross-feeding, autonomous, and switching populations, which reliably associated with the topology of the evolved metabolic networks as discussed in the main text. Increasing expression costs slowed down evolutionary dynamics, and two simulations (marked with an asterisk) fixed the topology of the metabolic network but did not both recruit both building block importers, and did not reach their final eco-evolutionary attractor within the duration of the simulation.

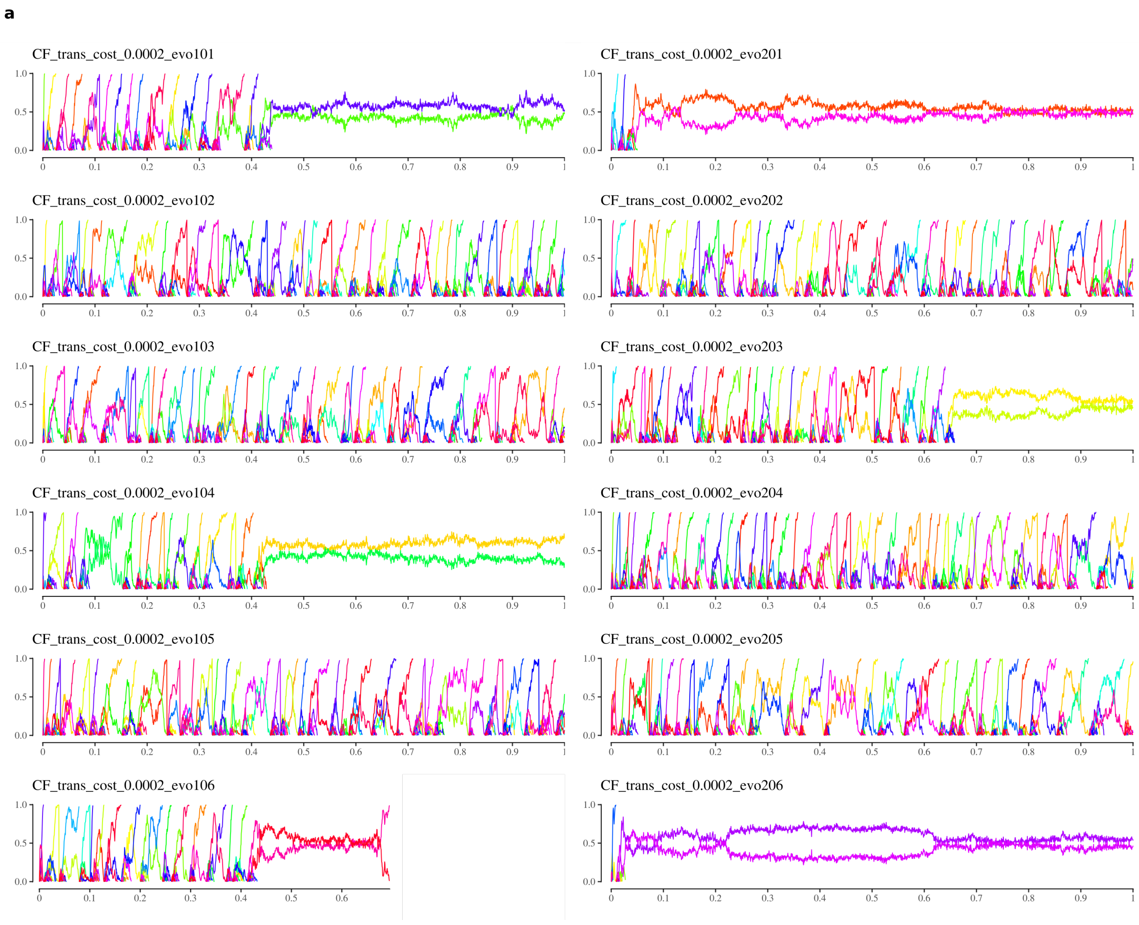

Figure 4: **Neutral lineage markers for 12 evolutionary simulations with reduced gene expression costs.**

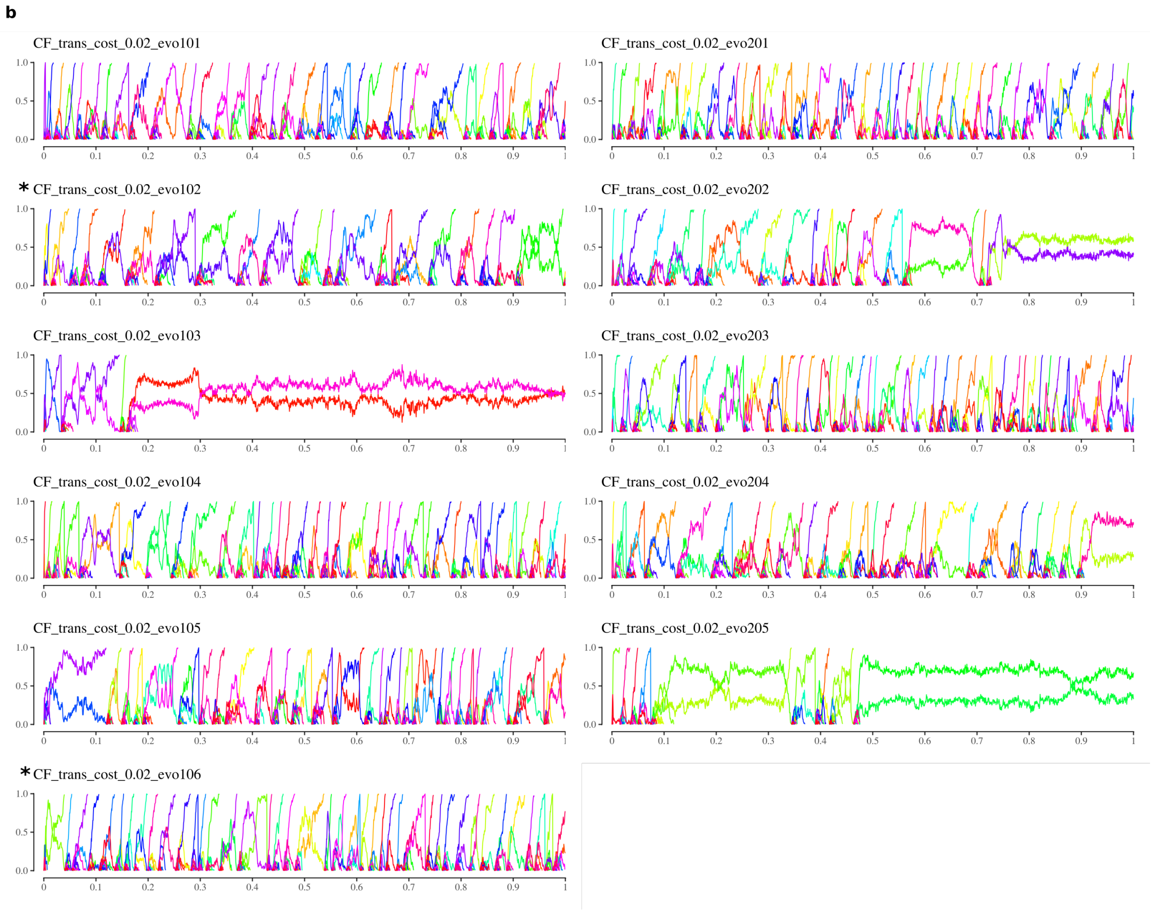

Figure 4: **(continued) Neutral lineage markers for 11 evolutionary simulations with increased gene expression costs**

1.5 Supplementary Figure 5: PCA of evolutionary trajectories of metabolic networks

For visualisation purposes we omitted populations 47 and 59 from the principal component analysis in the main text and Fig. 4a. We here show that omitting these two outlier populations had a negligible effect on the results: including them still separates the metabolic strategies along the first component and has a small effect on the proportions of variance and clustering of populations.

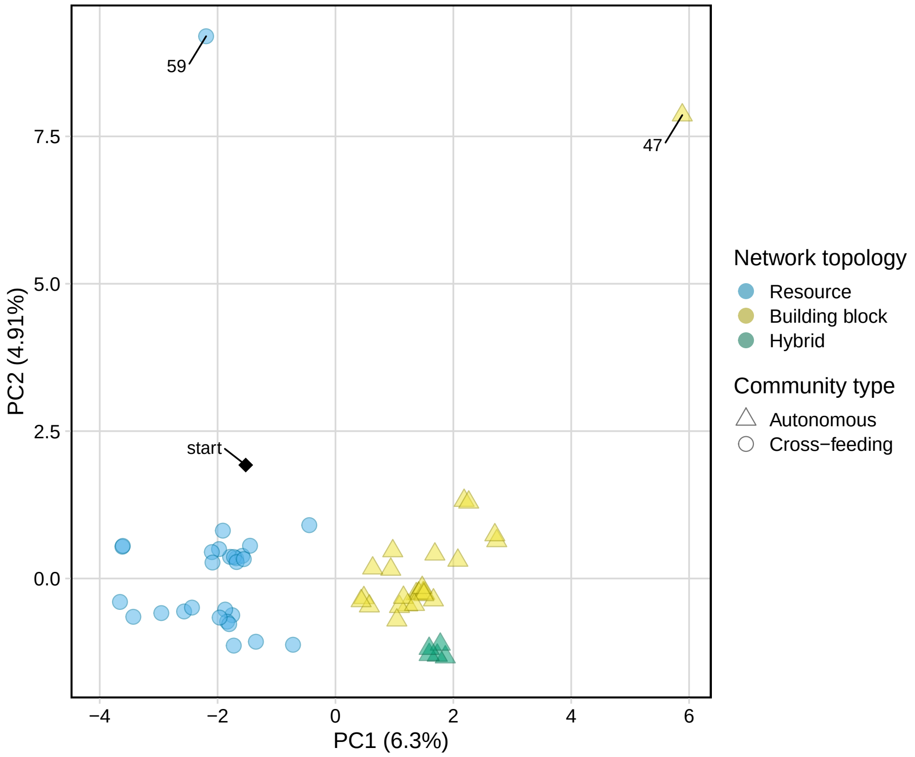

Figure 5: **PCA of evolutionary trajectories of metabolic networks** PCA of gene frequencies for 59 metabolic genes in 60 replicate populations during the whole main evolutionary experiment, including the two outlier populations (47 and 59). Only ancestral population and final time points are shown, see main text for details of analysis.

1.6 Supplementary Figure 6: Cross-feeding lineages self-organise into a spatially interleaved community

As discussed in the main text, the efficiency of cross-feeding was limited in spatially structured populations. Due to local reproduction cells continuously find themselves surrounded by their own conspecifics which do not (sufficiently) provide the necessary building blocks. As we also observed how the evolved cross-feeding lineages would form interleaved patterns in space, we thus asked the question: are two cross-feeding lineages more spatially dispersed than autonomous cells? To investigate this, we isolated two typical cross-feeding lineages from one of our evolutionary simulations, and one metabolically autonomous lineage from another simulation. When the system was initialised with the two cross-feeding genotypes spatially separated (see **Supplementary Fig. 6a**), the cells quickly self-organised into an interleaved mixture again (also see **Supplementary Movie 1**). We then did the same experiment with a single, metabolically autonomous genotype, which was coloured with two neutral markers. Interestingly, in the time span where the cross-feeding lineages had formed a mixed community, these metabolically autonomous cells remain spatially separated (see **Supplementary Fig. 6b**. Moreover, when these metabolically autonomous cells were initialised into a perfectly mixed state, the resulting pattern after some time was *less* dispersed than that of the two cross-feeding lineages. From this we conclude that cross-feeding communities are 'over-dispersed' in space.

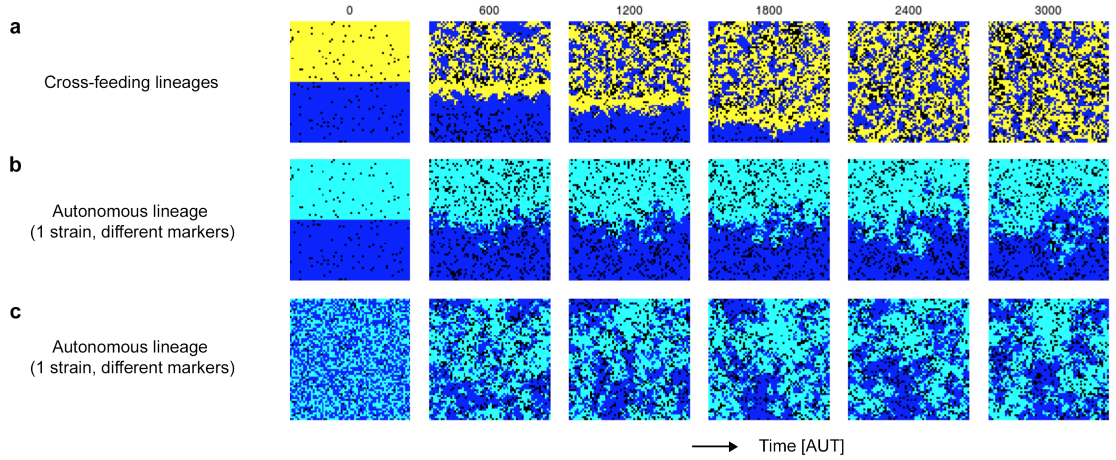

Figure 6: **Cross-feeding lineages self-organise into a spatially interleaved community**
a. Two genotypes isolated from a cross-feeding community quickly self-organise into an interleaved mixture.
b. One metabolically autonomous genotype, coloured with two different neutral markers, does not yield an interleaved pattern.
c. When one metabolically autonomous genotype is initialised perfectly mixed, it quickly results in a spatial distribution that is less mixed than the two cross-feeding lineages.
